# Diversified glucosinolate metabolism: biosynthesis of hydrogen cyanide and of the hydroxynitrile glucoside alliarinoside in relation to sinigrin metabolism in *Alliaria petiolata*

**DOI:** 10.3389/fpls.2015.00926

**Published:** 2015-10-31

**Authors:** Tina Frisch, Mohammed S. Motawia, Carl E. Olsen, Niels Agerbirk, Birger L. Møller, Nanna Bjarnholt

**Affiliations:** ^1^Plant Biochemistry Laboratory, Department of Plant and Environmental Sciences, University of CopenhagenCopenhagen, Denmark; ^2^VILLUM Research Center for Plant Plasticity, Department of Plant and Environmental Sciences, University of CopenhagenCopenhagen, Denmark; ^3^Center for Synthetic Biology “bioSYNergy”, Department of Plant and Environmental Sciences, University of CopenhagenCopenhagen, Denmark; ^4^Copenhagen Plant Science Center, Department of Plant and Environmental Sciences, University of CopenhagenCopenhagen, Denmark

**Keywords:** glucosinolates, alliarinoside, hydroxynitrile glucosides, *Alliaria petiolata*, biosynthesis, convergent evolution, HCN, chemical defense

## Abstract

*Alliaria petiolata* (garlic mustard, Brassicaceae) contains the glucosinolate sinigrin as well as alliarinoside, a γ-hydroxynitrile glucoside structurally related to cyanogenic glucosides. Sinigrin may defend this plant against a broad range of enemies, while alliarinoside confers resistance to specialized (glucosinolate-adapted) herbivores. Hydroxynitrile glucosides and glucosinolates are two classes of specialized metabolites, which generally do not occur in the same plant species. Administration of [UL-^14^C]-methionine to excised leaves of *A. petiolata* showed that both alliarinoside and sinigrin were biosynthesized from methionine. The biosynthesis of alliarinoside was shown not to bifurcate from sinigrin biosynthesis at the oxime level in contrast to the general scheme for hydroxynitrile glucoside biosynthesis. Instead, the aglucon of alliarinoside was formed from metabolism of sinigrin in experiments with crude extracts, suggesting a possible biosynthetic pathway in intact cells. Hence, the alliarinoside pathway may represent a route to hydroxynitrile glucoside biosynthesis resulting from convergent evolution. Metabolite profiling by LC-MS showed no evidence of the presence of cyanogenic glucosides in *A. petiolata*. However, we detected hydrogen cyanide (HCN) release from sinigrin and added thiocyanate ion and benzyl thiocyanate in *A. petiolata* indicating an enzymatic pathway from glucosinolates *via* allyl thiocyanate and indole glucosinolate derived thiocyanate ion to HCN. Alliarinoside biosynthesis and HCN release from glucosinolate-derived metabolites expand the range of glucosinolate-related defenses and can be viewed as a third line of defense, with glucosinolates and thiocyanate forming protein being the first and second lines, respectively.

## Introduction

**Figure 1:**
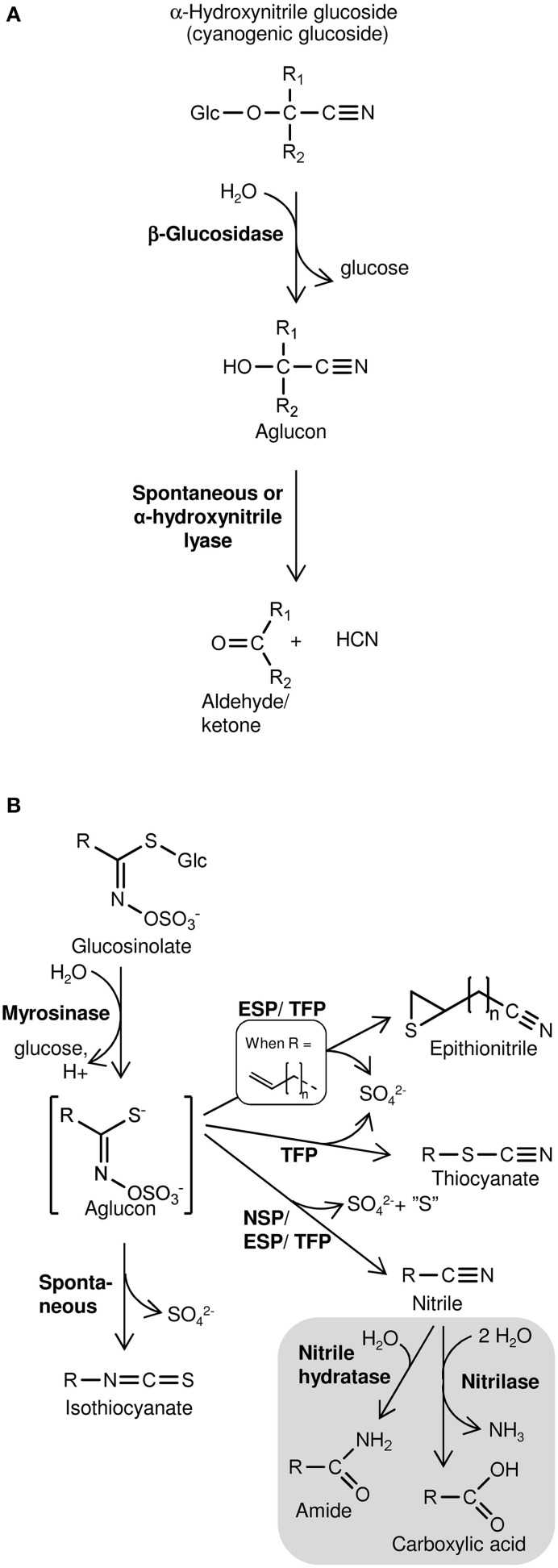
**Schematic overview of hydrolysis of cyanogenic glucosides (A) and glucosinolates (B) and subsequent product formation**. **(A)** Cyanogenic glucosides (α-hydroxynitrile glucosides) release HCN following hydrolysis of the β-glucosidic bond. In β- and γ-hydroxynitrile glucosides the CN-group is bound to another carbon than the O-glucosidic linkage. Consequently, hydrolysis of the β-glucosidic bond of these compounds does not result in HCN release. **(B)** Glucosinolate product formation is dependent on the variable side chain (R) structure and presence of specifier proteins (NSP, nitrile specifier protein; ESP, epithiospecifier protein; TFP, thiocyanate-forming protein). Gray section: Glucosinolate-derived nitriles may be metabolized *in vitro* by nitrilases possessing nitrilase activity and/or nitrile hydratase activity (Agerbirk et al., 2008). Nitrilase activity was suggested to mobilize nitrogen following glucosinolate turnover *in vivo* (Piotrowski, 2008).

Cyanogenesis, defined as HCN release, occurs from plants following β-glucosidase-catalyzed hydrolysis of cyanogenic glucosides (Figure 1A) (Morant et al., 2008; Gleadow and Møller, 2014). These α-hydroxynitrile glucosides are widespread defense compounds found in ferns, gymnosperms, and angiosperms, in some cases co-occurring with the structurally related non-cyanogenic β- and γ-hydroxynitrile glucosides (Bak et al., 2006; Bjarnholt and Møller, 2008). Amino acid-derived oximes are key intermediates in the biosynthesis of hydroxynitrile glucosides as well as glucosinolates, a group of sulfur- and nitrogen-containing specialized metabolites produced almost exclusively in the Brassicales (Halkier and Gershenzon, 2006; Bjarnholt and Møller, 2008; Takos et al., 2011). Extensive chemical diversity exist among glucosinolates and their degradation products produced upon hydrolysis of the thioglucosidic bond by myrosinases and subsequent conversion of the aglucon into isothiocyanates, simple nitriles, epithionitriles, or thiocyanates (Figure 1B) (Burow and Wittstock, 2009; Wittstock and Burow, 2010; Agerbirk and Olsen, 2012). The biologically inactive glucosinolates and hydroxynitrile glucosides and the activating hydrolytic myrosinases/β-glucosidases are generally separated by compartmentalization in intact tissue (Morant et al., 2008; Kissen et al., 2009). Disrupted compartmentalization and subsequent hydrolysis, which release toxic degradation products, is caused by tissue-damaging herbivores (Pentzold et al., 2013) or possibly by regulated physiological mechanisms in intact tissues. Evidence of such turnover of glucosinolates and hydroxynitrile glucosides in intact tissues indicate roles in defense against microbial pathogens (Bednarek et al., 2009; Clay et al., 2009; Møller, 2010). In addition, different routes of glucosinolate turnover including nitrilase-catalyzed hydrolysis of glucosinolate-derived nitriles may be involved in *in vivo* glucosinolate turnover with unknown biological significance (Figure 1B) (Falk et al., 2007; Piotrowski, 2008).

**Figure 2:**
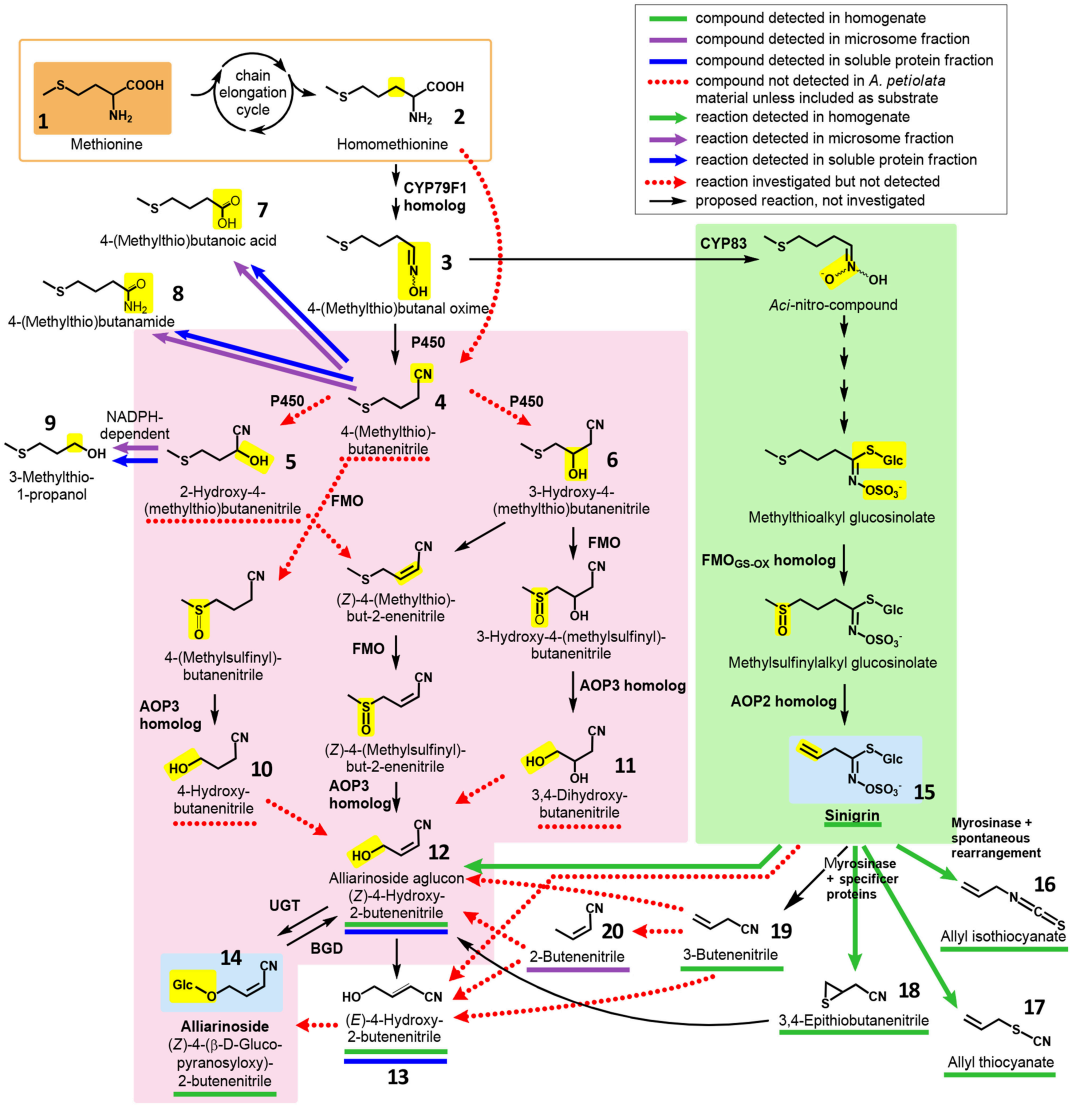
**Overview of hypothesized pathways and confirmed enzyme-catalyzed reactions in ***A. petiolata*** in relation to the biosynthesis of alliarinoside**. We previously suggested that alliarinoside (14) is biosynthesized from methionine (1) via a pathway (pink box) bifurcating from the sinigrin (15) pathway (green box) at the level of the homomethionine-derived oxime (3). In the present study, methionine (orange box) was confirmed as the amino acid precursor of alliarinoside. However, investigations of metabolites and enzyme activities in *A. petiolata* did not support the subsequent steps in the proposed pathway (pink box). Considering the structural resemblance of the aliphatic side chain of sinigrin and alliarinoside, we explored the alternative hypothesis that alliarinoside is biosynthesized via sinigrin-degradation. The alliarinoside aglucon, (Z)-4-hydroxy-2-butenenitrile (12), was confirmed to be sinigrin-derived. Further details of alliarinoside biosynthesis from sinigrin remain to be elucidated, but several options were tested. The box in the upper right corner explains color notations of documented presence of metabolites and enzyme-catalyzed conversions. Moieties resulting from the indicated enzymatic conversion are shown on a yellow background. Only compounds mentioned in the main text are numbered. CYP, cytochrome P450; AOP, antioxidant protein, in this case 2-oxoglutarate–dependent dioxygenase; FMO, flavin monooxygenase; UGT, UDP-glucosyl transferase; BGD, β-glucosidase.

Hydroxynitrile glucosides and glucosinolates rarely occur in the same plant species; only two exceptions are known. One is *Carica papaya* where the phenylalanine derived benzylglucosinolate co-occurs with minute amounts of the likewise phenylalanine derived cyanogenic glucoside prunasin (Olafsdottir et al., 2002). Another, more prominent, exception is *Alliaria petiolata* (garlic mustard, Brassicaceae), the only species known to contain glucosinolates, mainly sinigrin (15), as well as large quantities of a hydroxynitrile glucoside, alliarinoside (14) (structures shown in Figure 2), which acts as an insect feeding inhibitor (Haribal et al., 2001; Renwick et al., 2001). Furthermore, *A. petiolata* has been reported to release HCN after tissue disruption (Cipollini and Gruner, 2007) suggesting the presence of an unidentified cyanogenic glucoside. *A. petiolata* is native to Europa and parts of Asia and Africa, and is spreading invasively in North America (Cipollini et al., 2005). Alliarinoside and sinigrin may be key factors contributing to its success as an invader while the biological significance of HCN release is yet unknown (Frisch and Møller, 2012).

In a review based on the current knowledge about glucosinolate and hydroxynitrile glycoside biosynthesis in other species, a biosynthetic pathway for alliarinoside in *A. petiolata* bifurcating from sinigrin biosynthesis at the level of the oxime intermediate (3) was proposed (Figure 2, orange and pink sections) (Frisch and Møller, 2012). This hypothesis was incorrect. Instead, the biosynthetic studies reported here demonstrate that HCN release is derived from metabolism of sinigrin while alliarinoside is derived from methionine and may be derived from sinigrin turnover in intact tissue. This adds another dimension of complexity to the diverse roles of glucosinolate-derived products in plant defense.

## Results and discussion

### Alliarinoside is biosynthesized from methionine

Based on the chemical structure of alliarinoside (14), we hypothesized that this γ-hydroxynitrile glucoside is biosynthesized from methionine (1) via a pathway sharing the initial reactions of aliphatic chain elongation and oxime formation with the biosynthesis of sinigrin (15), a homomethionine-derived glucosinolate (Frisch and Møller, 2012) (Figure 2).

Methionine belongs to the amino acids derived from aspartic acid. Because [UL-^14^C]-methionine was not commercially available, biosynthetic studies of alliarinoside formation were initiated using [UL-^14^C]-aspartate as putative precursor. Formation of radiolabelled alliarinoside was indeed observed and incorporation via methionine was substantiated by the observed reduction of incorporation into alliarinoside in the presence of propargyl glycine (PAG), a specific inhibitor of methionine formation from aspartic acid (Figure S1).

**Figure 3:**
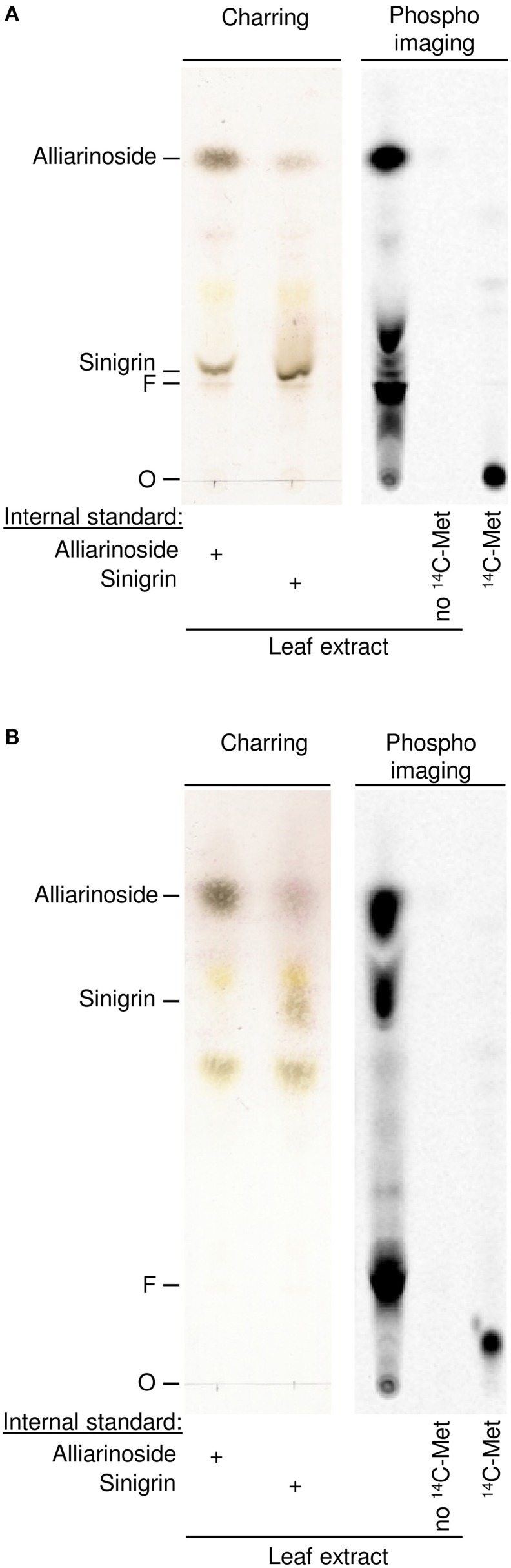
**Biosynthetic experiments documenting that alliarinoside (14) is methionine (1)-derived**. [UL-^14^C]-methionine was administered to *A. petiolata* leaves and formation of radiolabelled products was monitored by TLC using two different solvent systems to separate hydroxynitrile glucosides **(A)** and glucosinolates **(B)**, respectively. Representative results obtained with a single leaf are shown. The negatively charged sulfate group of sinigrin is prone to interact with the matrix and consequently cause different migration of sinigrin in the plant extract compared to an external standard. To more accurately determine the migration of alliarinoside and sinigrin in the plant extract, unlabeled internal standards were added to aliquots of leaf extract before samples were applied to the TLC plates (left panels). The spot showing increased staining following charring of the TLC plate indicated the position of the internal standard. O, origin; F, MeOH focus line.

Fortunately, a small amount of [UL-^14^C]-methionine was later obtained enabling us to test directly whether methionine is the amino acid precursor of alliarinoside. [UL-^14^C]-Methionine was administered to excised *A. petiolata* leaves through the petiole. After 24 h incubation in the light, methanol extracts of each leaf were analyzed by TLC (Figure 3). As observed in the experiments with [UL-^14^C]-aspartate, administration of [UL-^14^C]-methionine resulted in strong labeling of alliarinoside. Simultaneous strong labeling of sinigrin, a known methionine-derived glucosinolate, demonstrates that the [UL-^14^C]-methionine administered was taken up and efficiently incorporated in the sinigrin biosynthesis of the leaves.

### Systematic attempts to detect alliarinoside biosynthesis *In Vitro* via the previously suggested pathway were negative

Our first efforts to elucidate the route of alliarinoside biosynthesis from methionine were based on identification of putative intermediates in leaf homogenates of *A. petiolata* using GC-MS. The homogenate samples were prepared by suspending frozen leaf homogenate in ice cold buffer followed either by incubation or by immediate acidification in 1 M HCl and extraction in CH_2_Cl_2_ (*t* = 0), thereby quenching the majority of enzymatic activities. Originally, it was hypothesized that the alliarinoside pathway branched off from the sinigrin pathway at the oxime derived from homomethionine (2), 4-(methylthio)butanal oxime (3), and proceeded via 4-(methylthio)butanenitrile (4) (Figure 2, pink box). Search by GC-MS for compounds with masses corresponding to the proposed intermediates was unsuccessful with exception of the alliarinoside aglucon, (*Z*)-4-hydroxy-2-butenenitrile (12), which was detected in large amounts along with minor amounts of the E-isomer, (*E*)-4-hydroxy-2-butenenitrile (13) (Figure S2). An apparent decrease in the detectable amount of alliarinoside aglucon was observed following incubation of the homogenate. This indicated further metabolism of the aglucon although the biological variation was substantial (Figure S3A). The variation is presumably caused by a combination of a strong variation in initial alliarinoside content as previously demonstrated (Frisch et al., 2014) and our inability to completely quench all enzymatic activity in the *t* = 0 samples, possibly causing β-glucosidase mediated conversion of some of the alliarinoside pool in these control samples. The possible product formed from further metabolism of the alliarinoside aglucone remains unidentified, but may be the derived furanone (γ-lactone) formed by hydrolysis of the nitrile group and subsequent cyclization of the hydroxyacid as previously suggested, resulting in antifungal and antimicrobial defense (Bjarnholt and Møller, 2008; Frisch and Møller, 2012; Saito et al., 2012).

A different approach was taken to study the possible involvement of some of the intermediates in the suggested pathway. The proposed intermediates were purchased or chemically synthesized and tested as putative substrates in enzyme activity assays using either dialyzed microsomal preparations or desalted soluble protein extracts isolated from young rosette *A. petiolata* leaves. Aliquots of the protein preparations were incubated with putative substrates in the absence or presence of a series of putative enzyme cofactors. These included NADPH as a cofactor for cytochrome P450s (P450s) (Sibbesen et al., 1995; Hannemann et al., 2007) and flavin monooxygenases (FMOs) (Hansen et al., 2007; Li et al., 2008), and 2-oxoglutarate, ascorbate, and ferrous ion (FeSO_4_) administered as cofactors for 2-oxoglutarate-dependent dioxygenases (2-ODDs), which includes the AOP3 homolog suggested to be involved in the pathway (De Carolis and De Luca, 1993; Kliebenstein et al., 2001; Frisch and Møller, 2012). Furthermore, catalase was added to reduce the level of reactive oxygen species, which may inhibit 2-ODDs (Britsch and Grisebach, 1986; Lange et al., 1994). Substrate conversion and product formation was monitored by GC-MS-analysis of trimethylsilyl (TMS)-derivatized samples. The quantity of a given compound was calculated relative to the internal standard (benzonitrile, *m*/*z* 103) based on the peak areas in the extracted ion chromatograms (EICs).

4-(Methylthio)butanenitrile (4) was hypothesized as the first committed intermediate in the alliarinoside pathway, but this putative intermediate was not produced by microsomes incubated with homomethionine (3) and NADPH (Figure 2). Assuming that 4-(methylthio)butanenitrile was nevertheless formed *in vivo*, we searched for possible enzymatic conversion products in various samples. Hydroxylation might result in formation of either of the 2- or 3-hydroxyl derivatives (5 and 6, respectively), but none of these compounds were detected in leaf homogenates. Investigations of metabolism of 4-(methylthio)butanenitrile (4) or 2-hydroxy-4-(methylthio)butanenitrile (5) by microsomes and soluble proteins showed enzymatic turnover of the substrates but no production of any of the suggested products (5, 6 and un-numbered compounds in pink box in Figure 2) (Figures S4, S5). The detected enzyme activities and products (compounds 7, 8, 9) (Figures S4, S5) (Figure 2, top, white background) were considered unlikely to be involved in alliarinoside biosynthesis.

Focusing on the last part of the hypothetical alliarinoside pathway (Figure 2, pink box), 4-hydroxybutanenitrile (10) or 3,4-dihydroxybutanenitrile (11) would represent likely immediate biosynthetic precursors of the alliarinoside aglucon (12), which we had observed in the homogenate. However, in three independent experiments with soluble protein preparations no evidence of enzymatic conversion of these potential precursors into the aglucon or its (*E*)-isomer (13) could be demonstrated as monitored by GC-MS-analysis. On the other hand, small amounts of the aglucon and minute amounts of its (*E*)-isomer were detected in the desalted soluble protein preparation without any substrate addition. The low amounts of these (*Z*)- and (*E*)-isomers increased five and two-fold, respectively, when the soluble protein preparation was supplemented with a cofactor mixture containing NADPH, 2-oxoglutarate, ascorbate, and catalase (Figure S6). This demonstrated that the desalted soluble protein preparation had retained a residual endogenous, unidentified precursor from which it was able to produce small amounts of the alliarinoside aglucon (and minute amounts of the (*E*)-isomer) in a cofactor-dependent manner. Supplementation of the cofactors with ferrous iron, required by some 2-ODDs (Prescott, 2000), did not further increase product formation (Figure S6). The overall conclusion from these experiments is that although we observed low biosynthesis of the alliarinoside aglucon *in vitro* from trace amounts of an unidentified compound, we did not find evidence supporting our originally proposed biosynthetic pathway from a sinigrin precursor, 4-(methylthio)butanal oxime (3) (Figure 2, pink box). Consequently, we considered whether a sinigrin product could instead lead to alliarinoside (Figure 2, green box).

### *In Vitro* formation of the alliarinoside aglucon from sinigrin

**Figure 4:**
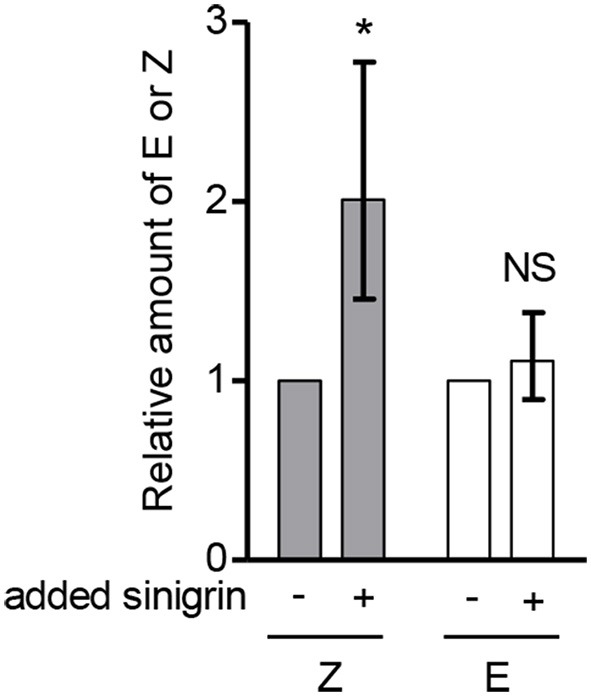
**Addition of sinigrin (15) to the myrosinase-containing homogenate of ***A. petiolata*** results in formation of the alliarinoside aglucon (12)**. The relative content of the alliarinoside aglucon (Z) and much lower content of its (*E*)-isomer (E) (13) in leaf homogenates following incubation with or without exogenously added sinigrin. Statistical analyses were performed on log_10_-transformed ratios (*n* = 3). To ease visual interpretation, the back-transformed mean ± standard deviation (SD) is shown. Accordingly, the SD is asymmetric. The one-tailed paired *t*-test showed a significant increase (^*^P < 0.05) of the aglucon formed in the presence of exogenously added sinigrin compared to untreated samples. NS, not significant.

Considering that sinigrin (15) is a major metabolite in *A. petiolata*, has a C4 aglucon similar to alliarinoside (14) and is biosynthesized from methionine (3), we explored whether alliarinoside was derived from metabolism of this glucosinolate. To test the hypothesis that the alliarinoside aglucone (12) could be produced from sinigrin, samples were spiked with sinigrin and incubated to enable metabolic turnover. We saw no activity when using desalted protein fractions with additions of the previously mentioned co-factors, hence the reported results originate from leaf homogenate samples. Analysis by GC-MS of TMS-derivatized samples revealed the presence of large amounts of the alliarinoside aglucon, (*Z*)-4-hydroxy-2-butenenitrile (12). Homogenate incubated without exogenously added sinigrin (15) also contained considerable amounts of alliarinoside aglucon. To determine whether the content of the alliarinoside aglucon was significantly increased following incubation with exogenous sinigrin in comparison to the content in the untreated homogenate, the experiment was performed three times with different batches of leaf homogenate. In each experiment, the EIC peak area representing (*Z*)-4-hydroxy-2-butenenitrile relative to that of the internal standard was determined. As the starting materials were different a direct comparison of absolute amounts was not possible, so for each experiment the ratio between technical replicate means of sinigrin-spiked and untreated samples was calculated. The variation between experiments was still great, as seen from the standard deviation in Figure 4. In all experiments however, the calculated ratio was > 1. To test the hypothesis that the (*Z*)-4-hydroxy-2-butenenitrile content increased when leaf homogenate was incubated with singrin a one-tailed paired *t*-test was carried out. As ratio data do not follow a normal distribution, the ratios were log_10_-transformed to normalize the positive skewness. A one-tailed paired *t*-test of the transformed data showed a significant increase in (*Z*)-4-hydroxy-2-butenenitrile content following sinigrin addition (*P* = 0.0325). In contrast, the amount of the (*E*)-isomer (13) did not increase significantly. Assuming linearity and equal signal intensity of (*Z*)-4-hydroxy-2-butenenitrile and the (*E*)-isomer standard, the stoichiometric recovery of the exogenously applied sinigrin in (*Z*)-4-hydroxy-2-butenenitrile was highly variable ranging between 0.6 and 33%, most likely reflecting variation in initial concentrations of both sinigrin and alliarinoside as well as biosynthetic activity and the previously demonstrated further metabolic conversion of the aglucon (Figure S3). This experiment confirmed the hypothesis that sinigrin is a precursor of (*Z*)-4-hydroxy-2-butenenitrile, the aglucon of alliarinoside (Figure 2).

**Figure 5:**
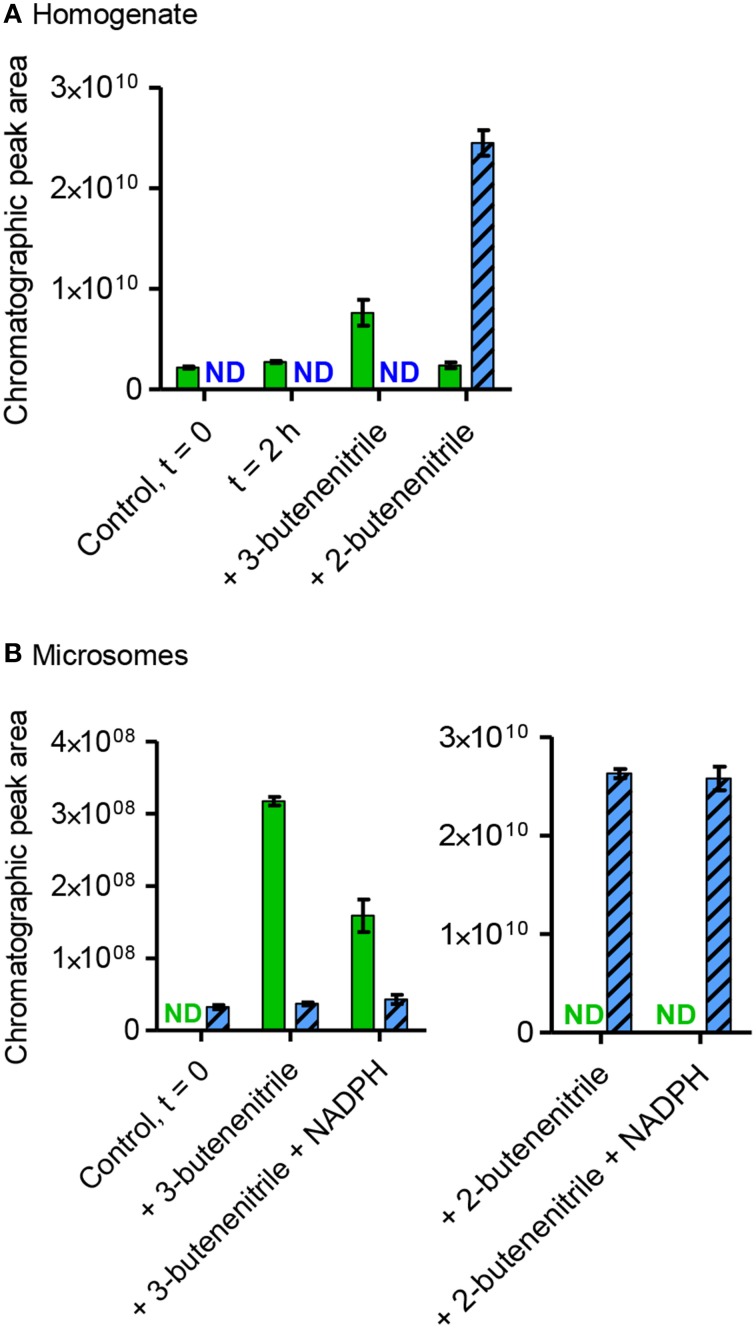
**Presence, production and consumption of 3-butenenitrile (19) and 2-butenenitrile (20) following incubation with homogenate or microsomes from ***A. petiolata*****. Homogenate **(A)** or microsomes **(B)** were incubated with or without addition of exogenous 3-butenenitrile or 2-butenenitrile (mixture of (*Z*)- and (*E*)-isomers), homogenate for 2 h and microsomes for 30 min. The content of 3-butenenitrile (green bars, *m*/*z* 67, 4.2 min) and 2-butenenitrile (blue striped bars, *m*/*z* 67, sum of isomers: 3.8+4.5 min) at the end of the incubation period was determined as the EIC peak area as obtained by SPME-GC-MS. The mean ± range of two technical replicates is depicted. For homogenate the incubated unspiked control is shown to evaluate whether endogenous sinigrin in this fraction led to an increase in the amounts of 3- or 2-butenenitrile. ND, not detected; *t* = 0, time point zero.

GC-MS-based investigations were performed to identify potential intermediates in the conversion of sinigrin to alliarinoside. Allyl isothiocyanate (16), allyl thiocyanate (17), and 3,4-epithiobutanenitrile (18) were present in leaf homogenates and the amounts increased significantly following sinigrin addition, but also in this case with substantial biological variation in the amounts formed of the individual species (Figure S7). The simple nitrile (3-butenenitrile, 19) was not detectable at the conditions used, but using solid phase microextraction (SPME)-GC-MS we detected the presence of 3-butenenitrile in the leaf homogenate and 2-butenenitrile (20) in the microsomal fraction (Figure 5). As absorption onto the SPME fiber is not quantitative, no absolute measures could be calculated, and thus these samples were not spiked with sinigrin. When unspiked homogenate was incubated there was no evident increase in the concentration of 3-butenenitrile, the simple nitrile expected to be formed from the sinigrin present in this fraction (Figure 5A). This could be either because the increase was not sufficient to be detected by SPME-GC-MS, or because the nitrile was further metabolized. Due to the evident structural resemblance between the detected 2- and 3-butenenitrile and the alliarinoside aglucon (12) we tested these as potential intermediates in alliarinoside biosynthesis. In the microsomal fraction exogenously added 3-butenenitrile appeared to decrease substantially when NADPH was present, while 2-butenenitrile appeared to not be metabolized (Figure 5B). However, no formation of the alliarinoside aglucon or the (*E*)-isomer (13) was detected following addition of 3- or 2-butenenitrile to homogenate or microsomes supplemented with NADPH (Figure 2). Neither was 2-butenenitrile detected as a product from 3-butenenitrile metabolism (Figure 5). In contrast, 3-butenenitrile was found to be converted into 3-butenoic acid in homogenate and the microsomal fraction, which indicated the presence of nitrilase activity (Figure S8). Formation of the alternative nitrilase product, 3-butenamide, was not observed, but exogenously added 3-butenamide was metabolized into 3-butenoic acid by leaf homogenate indicating the presence of amidase activity. However, these conversions appear as unlikely events in sinigrin-derived alliarinoside formation.

**Figure 6:**
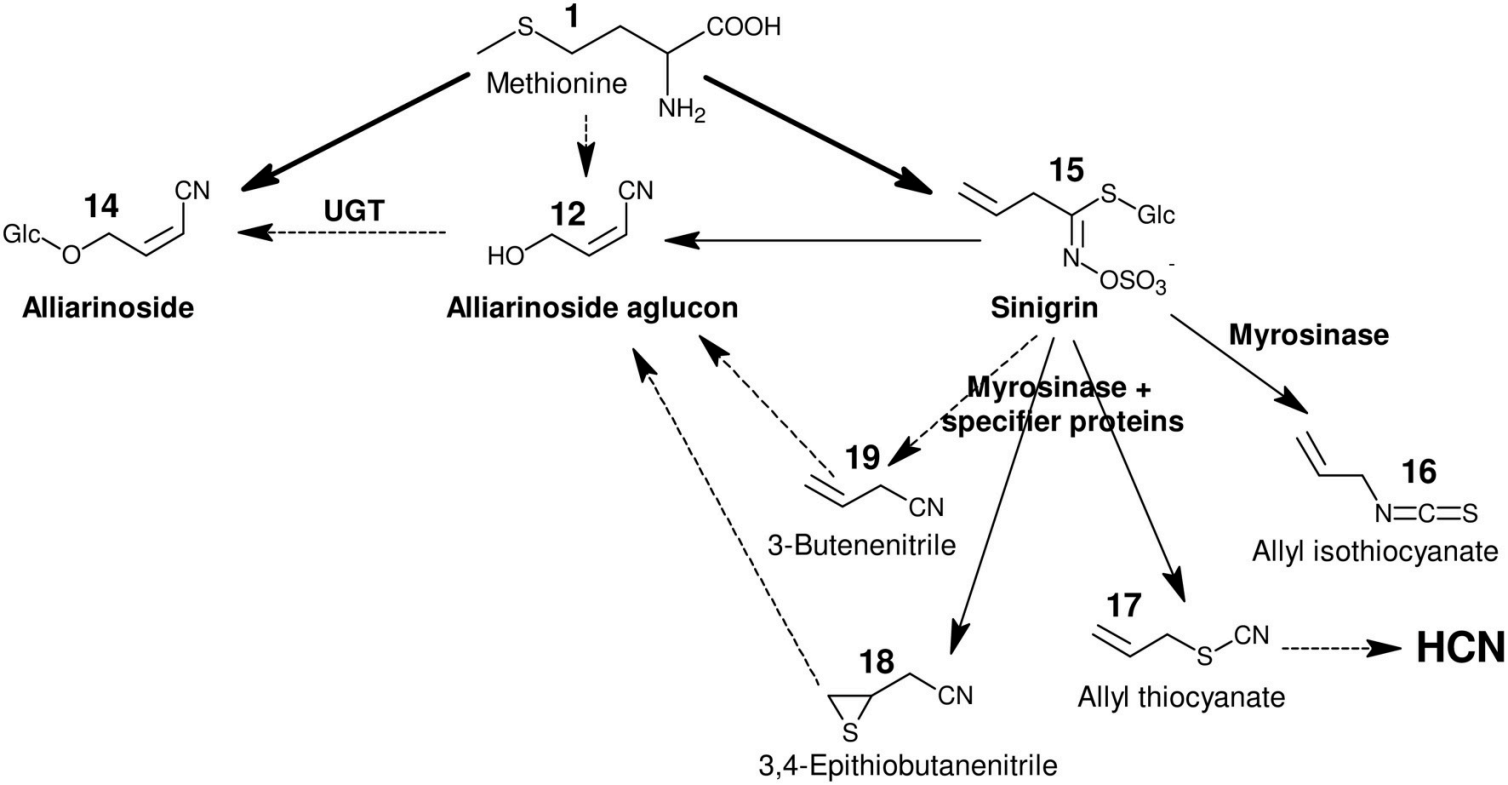
**Proposed biosynthetic origin of alliarinoside and HCN in ***A. petiolata*****. All illustrated metabolites were detected in either homogenate or microsomes prepared from *A. petiolata* leaves. Bold arrows signify biosynthesis demonstrated *in planta*, plain solid arrows signify reactions demonstrated *in vitro*, and dotted arrows indicate proposed reactions based on these reactions and other results obtained in the current study. The alliarinoside aglucon (12) is presumed to be produced by only one of the proposed routes *via* either 3,4-epithiobutanenitrile (18) or 3-butenenitrile (19). For further details on tested reactions, occurrence of compounds and abbreviations refer to Figures 2–5 regarding alliarinoside and Figures 7, 8 regarding HCN.

Figure 6 summarizes the positive results obtained in this study and illustrates the proposed biosynthetic origin of alliarinoside based on these results. In conclusion, sinigrin (15) is a precursor of the alliarinoside aglucon (12), at least *in vitro*. Sinigrin breakdown also produced the derived epithionitrile (18), simple nitrile (19), thiocyanate (17), and isothiocyanate (16). Considering the presence of a nitrile group, 3-butenenitrile (19) and 3,4-epithiobutanenitrile (18) are structurally more likely precursors of alliarinoside than the other detected sinigrin degradation products. Various routes from 3-butenenitrile to the alliarinoside aglucon (12) can be envisioned: 1. Initial isomerization to 2-butenenitrile (20) followed by hydroxylation at the C4-position by a P450, FMO, or dioxygenase, or 2. Initial epoxidation and hydrolysis of the double bond to afford a diol, followed by elimination of one hydroxyl group yielding the 4-hydroxy alkene nitrile. The detection of 2-butenenitrile in the dialyzed microsomal fraction may indicate tight association of this metabolite to membrane-associated proteins or embedment in the lipid bilayer, but its potential involvement in alliarinoside biosynthesis was not confirmed, and neither was any involvement of 3-butenenitrile. 3,4-Epithiobutanenitrile was not available for experimental testing, but hydrolysis to 4-hydroxy-3-sulfanylbutanenitrile (HOCH_2_-CHSH-CH_2_-CN) followed by loss of H_2_S would be a third likely pathway to the alliarinoside aglucon. This suggestion would seem to be in accordance with the recent discovery in *A. petiolata* of a glucoside, petiolatamide, with structural resemblance to the simple unsaturated nitrile from sinigrin, 3-butenenitrile (Frisch et al., 2014). Alliarinoside could be derived from the epithionitrile (18), with 3-butenenitrile (19) produced in parallel being converted to petiolatamide. Besides the common sinigrin degradation products, no other detected metabolites than the alliarinoside aglucon increased significantly after sinigrin addition to the leaf homogenate. The intermediates in the pathways of glucosinolate core structure and cyanogenic glucoside formation are highly channeled (Møller and Conn, 1980; Jørgensen et al., 2005; Sønderby et al., 2010). If this is also the case for alliarinoside biosynthesis, identification of the intermediates may prove challenging as illustrated by the elusive product of the observed NADPH-dependent increase in 3-butenenitrile metabolism. In similar *in vitro* investigations of enzymatic activities in *L. japonicus* Saito et al. (2012) were able to detect production of the presumed intermediate nitrile in the production of a γ-hydroxynitrile glucoside, but not the hydroxylation reaction leading to the aglucon of this compound. Furthermore, in the homogenates and protein preparations used, the compartmentalization of the glucosinolates and the enzymes involved in their degradation has been disrupted, and this *in vitro* situation may obviously result in a product profile different from that in intact tissue. This different product profile likely includes the observed NADPH-independent nitrilase activity converting 3-butenenitrile to 3-butenoic acid, which supports *in vitro* evidence from other Brassicaceae members suggesting metabolism of glucosinolate-derived nitriles by nitrilases evolved in this family (Janowitz et al., 2009).

### *A. Petiolata* does not contain cyanogenic glucosides, but converts glucosinolate-derived thiocyanates into HCN

To investigate whether cyanogenic glucosides were present in *A. petiolata*, metabolite profiling of different developmental stages of *A. petiolata* leaves from Danish populations was performed by LC-MS. When analyzed by our system of electrospray ion trap MS with sodium ions present in the mobile phase, cyanogenic glucosides display a diagnostic loss of HCN (*m*/*z* = M + Na–27), which does not arise from β- and γ-hydroxynitrile glucosides. In addition, glucosides display a characteristic fragment ion of *m*/*z* = 185 ([Glc+Na-H_2_O]^+^) (Bjarnholt et al., 2008). We combined LC-MS analyses of authentic standards of several cyanogenic glucosides with search in *A. petiolata* extracts for glucosides losing HCN. These attempts did not provide any indication of the presence of cyanogenic glucosides in *A. petiolata* (Figure S9).

To confirm the reported cyanide release from *A. petiolata* (Cipollini and Gruner, 2007), the HCN potential in Danish populations was determined. HCN can be detected by various colorimetric assays as described in this section, but a number of compounds can cross-react in these assays. Therefore, the volatile HCN is allowed to diffuse out of the sample to be trapped in an alkaline trap solution and thus separated from the interfering compounds in the sample solution. In the current case, HCN released after tissue grinding and trapped in an alkaline trap was quantified in a colorimetric assay modified from König's synthesis of pyridine dyes (König, 1904; Lambert et al., 1975). Young rosette leaves of *A. petiolata* showed a color response equivalent to a low HCN potential of 0.4 ± 0.02 nmol mg FW^−1^ (mean ± SD, *n* = 3).

**Figure 7:**
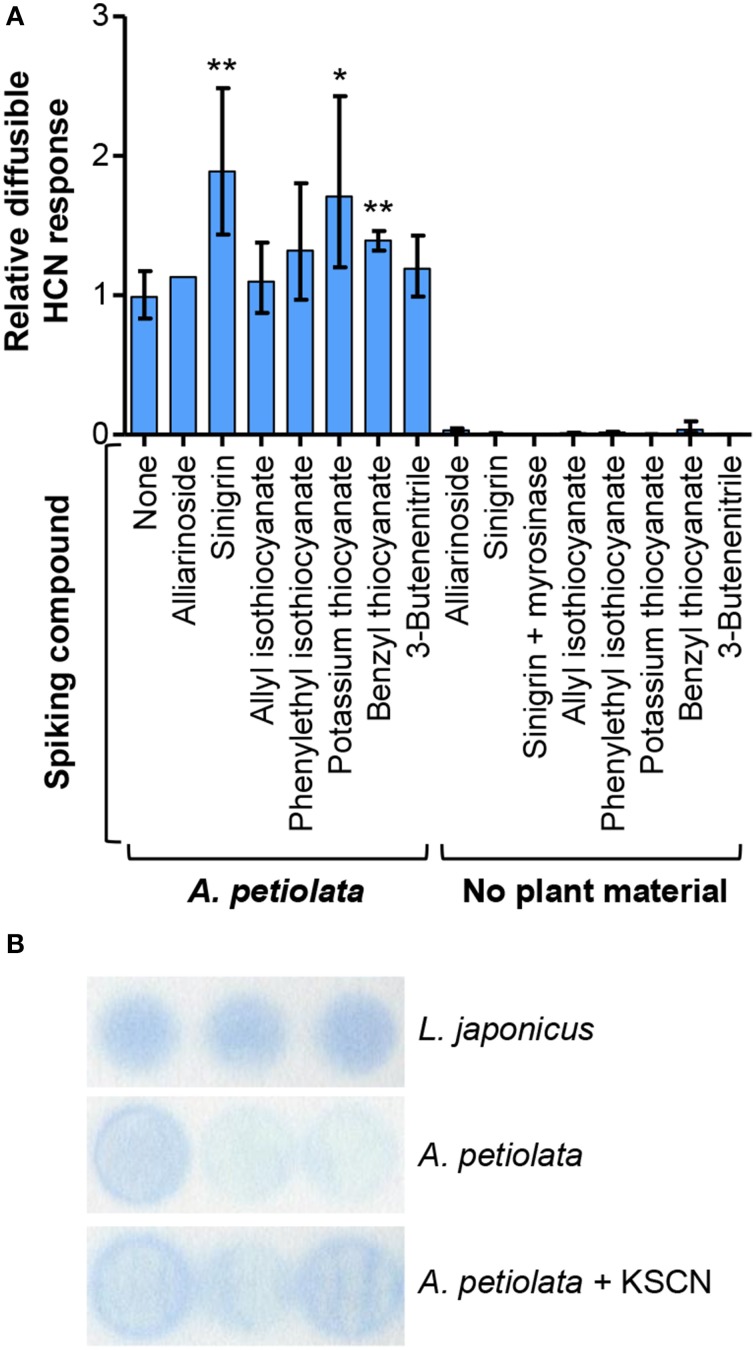
**The diffusible HCN response from sinigrin and organic/inorganic thiocyanate is dependent on the presence of ***A. petiolata*** enzymes**. **(A)** Colorimetric HCN detection based on the König reaction was used to quantify the diffusible HCN response in homogenates of *A. petiolata* leaves. The effect of spiking the homogenate with alliarinoside (14), sinigrin (15) or available glucosinolate degradation products was quantified relative to the untreated samples (*n* = 4, except alliarinoside: *n* = 2). The relative diffusible response was significantly increased by addition of sinigrin, benzyl thiocyanate (^**^P < 0.01) or KSCN (^*^P < 0.05), whereas other compounds had no significant effect (one-tailed unpaired *t*-test of log_10_-transformed ratios). The back-transformed mean ± SD is depicted. **(B)** Feigl-Anger paper, a different colorimetric method for HCN detection, was used to confirm the effect of KSCN on the diffusible response from leaf homogenates of *A. petiolata*. Results of triplicates are shown. As positive control, highly cyanogenic *Lotus japonicus* leaf material was used (4 mg FW sample^−1^). Larger amounts of leaf material were applied to obtain a detectable response from *A. petiolata* (60 mg FW sample^−1^). There was a tendency to more intense color development in *A. petiolata* samples added KSCN.

Efforts to identify the chemical source of this diffusible response in the HCN assay were made by investigating the effect of spiking *A. petiolata* leaf homogenate with alliarinoside, sinigrin, or glucosinolate degradation products. Sinigrin is the dominant glucosinolate in *A. petiolata*, and unfortunately not all sinigrin degradation products were available as standards. In lack of allyl thiocyanate (17), the effects of benzyl thiocyanate and KSCN (Figure 7) were tested. The spiking concentrations were comparable to the endogenous levels of alliarinoside and sinigrin (Figure S10). Sinigrin and thiocyanates significantly increased the diffusible response, whereas alliarinoside had no effect (Figure 7A). Neither isothiocyanate derived from sinigrin (16) or phenethyl glucosinolate, nor the simple nitrile from sinigrin, 3-butenenitrile (19), increased the diffusible response. These studies imply that the diffusible HCN response from *A. petiolata* arises from sinigrin *via* degradation to allyl thiocyanate. In absence of *A. petiolata* material, no volatile HCN response was detected from sinigrin, myrosinase-treated sinigrin, or any of the tested thiocyanates. Thus, the observed response requires additional conversion of organic and inorganic thiocyanate. This reaction is not restricted by the alkyl group of the organic thiocyanate, as inorganic, or aromatic thiocyanate as well as aliphatic sinigrin was found to increase the response. As shown in Figure 7B, the diffusible HCN response from *A. petiolata* and the effect of KSCN addition was confirmed using a different colorimetric HCN detection method, the Feigl-Anger assay (Feigl and Anger, 1966; Takos et al., 2010), supporting that the detected compound was HCN. Thiocyanate ion (SCN^−^) can cross-react in the König reaction (Sharma and Thibert, 1985) and possibly also with the Feigl-Anger paper (Brinker and Seigler, 1992). Indeed, both tested thiocyanates gave a non-volatile response independent of *A. petiolata* (Figure S11). The diffusion step was thus included when using both detection systems which completely eliminated this cross-reaction (Figure 7A), as protonation and thus volatilization of SCN^−^ was negligible at the applied pH 6 (HSCN pK_a_ = 0.9; HCN pK_a_ = 9.2). Inorganic thiocyanate ion is a well-established hydrolysis product of indole glucosinolates that have indeed been reported from *A. petiolata* leaves (Agerbirk et al., 2010).

**Figure 8:**
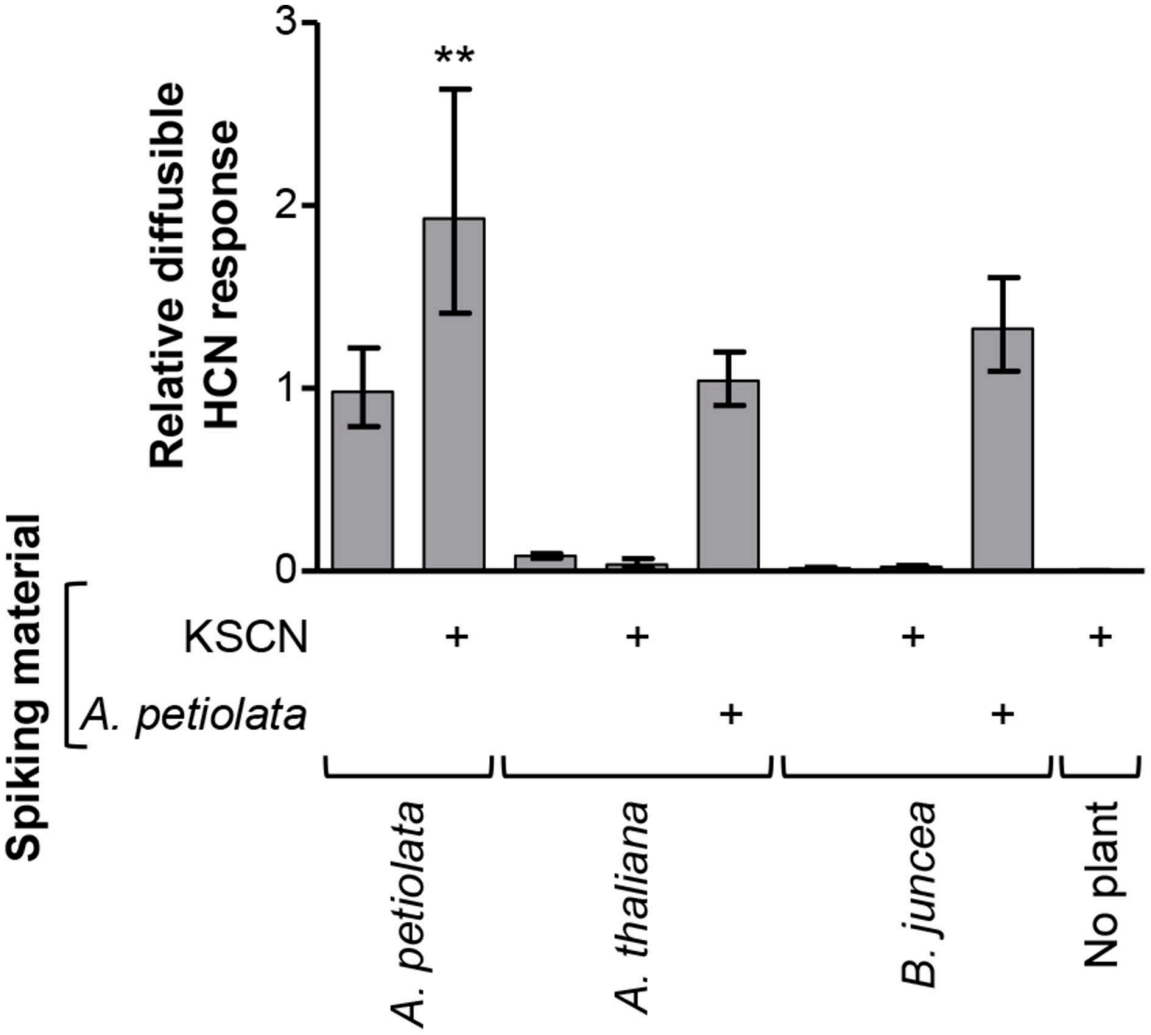
**The glucosinolate-derived diffusible HCN response is not general to the Brassicaceae**. *A. thaliana* and *B. juncea* were investigated for their ability to provide a diffusible HCN response from endogenous glucosinolates or from exogenously added KSCN. The response in the König reaction-based diffusion assay was quantified relative to the mean of unspiked *A. petiolata* samples and a one-tailed unpaired *t*-test was performed on the log_10_-transformed ratios. Bars represent back-transformed mean ± SD. (^**^P < 0.01; *n* = 4).

To determine whether the thiocyanate-derived diffusible response in the HCN assays is common for glucosinolate producers, two other Brassicaceae species were analyzed. *Brassica juncea* and *Arabidopsis thaliana* Col-0 were selected for their presence and absence of sinigrin, respectively, which was confirmed by LC-MS analysis (Figure S10). When analyzed in the König reaction-based assay, neither of these species gave a KSCN-derived diffusible HCN response (Figure 8). Presence of *A. thaliana* material neither inhibited nor increased the response from *A. petiolata* when material from the two plant species was combined. Blends of *B. juncea* and *A. petiolata* yielded a small but insignificant increase in the diffusible response, compared to *A. petiolata* alone (*P* = 0.06), possibly due to conversion of *B. juncea* sinigrin by *A. petiolata*. In conclusion, the observed sinigrin- and thiocyanate-derived diffusible HCN response from *A. petiolata* is not a common feature of glucosinolate-producing plants and does not arise from the presence of cyanogenic glucosides in *A. petiolata*.

Methyltransferases in *A. thaliana* Col-0 and *Brassica oleracea* methylate the SCN ion resulting in antibacterial, volatile CH_3_SCN (Attieh et al., 2002; Nagatoshi and Nakamura, 2009). However, no KSCN-derived diffusible response was observed from *A. thaliana* Col-0 in the present study (Figure 8), which demonstrates that CH_3_SCN did not cross-react in our experimental setup with the alkaline trap. Thus, though homologs of the methyltransferase in *A. thaliana* Col-0 are present across the plant kingdom (Nagatoshi and Nakamura, 2009) and may be involved in volatilization of SCN^−^ in *A. petiolata*, CH_3_SCN can be excluded to have cross-reacted in the two applied types of HCN diffusion assay. Hence, we conclude that the observed diffusible response in the HCN assays was caused by HCN produced by *A. petiolata* from glucosinolate-derived organic or inorganic thiocyanate. Sinigrin is the dominant glucosinolate in *A. petiolata* and allyl thiocyanate was detected as a sinigrin-degradation product in *A. petiolata* (Figure S7). This strongly supports that sinigrin *via* allyl thiocyanate is the main endogenous precursor of HCN in disrupted *A. petiolata* leaf tissue (Figure 6). Indole glucosinolate concentrations are very low in *A. petiolata* leaves but quite high in floral parts (Agerbirk et al., 2010). Hence, both allyl thiocyanate and inorganic thiocyanate ion could be *in vivo* intermediates in HCN release from *A. petiolata*.

Several sinigrin-derived degradation products were detected in *A. petiolata* extracts and thus not all sinigrin was converted into allyl thiocyanate upon hydrolysis. This may partly explain the observed 12-fold molar difference in sinigrin leaf concentration (5 nmol mg FW^−1^) and the diffusible HCN response (0.4 nmol mg FW^−1^). Interestingly, the diffusible HCN response increased less than two-fold, when *A. petiolata* homogenate was added 10 nmol mg FW^−1^ of KSCN or benzyl thiocyanate. This indicates that the response was not limited solely by the thiocyanate concentration: further conversion of thiocyanate was also a limiting factor. The mechanism of this thiocyanate processing remains to be elucidated, but the reaction was dependent on the presence of *A. petiolata* homogenate, which strongly suggests an enzymatic reaction.

### Biology of the HCN release

The thiocyanate processing yielding HCN from *A. petiolata* may be a trait associated with the presence of TFP activity enabling thiocyanate formation upon glucosinolate hydrolysis. TFPs are relatively rare, more recently evolved, specifier proteins in Brassicaceae (Kuchernig et al., 2012), and the catalytic mechanism allows only certain glucosinolates to act as substrates for these enzymes (Rossiter et al., 2007; Brandt et al., 2014). ApTFP1 was recently identified in *A. petiolata* (Kuchernig et al., 2012), whereas no TFP is present in *A. thaliana* Col-0, which predominantly produces isothiocyanates from glucosinolate degradation (Burow and Wittstock, 2009) and thiocyanate ion from indole glucosinolates, although the latter is formed in a spontaneous reaction, not mediated by TFP (Agerbirk and Olsen, 2012). To our knowledge, no organic thiocyanates have been detected in *B. juncea*, which was reported to produce the isothiocyanate and small amounts of the epithionitrile from sinigrin (Cole, 1976). It remains an open question whether the observed thiocyanate-derived HCN release is a trait evolved in *A. petiolata* or is present in other TFP-containing species, e.g., the closely related *Thlaspi arvense* (Beilstein et al., 2006; Kuchernig et al., 2011).

The observed diffusible HCN response from *A. petiolata* was very low compared to reported values of HCN release from leaves of cyanogenic glucoside-containing plants (Lechtenberg and Nahrstedt, 1999). HCN is toxic to various organisms, but many pathogens and herbivores have evolved countermeasures to overcome the toxicity (Gleadow and Woodrow, 2002; Gleadow and Møller, 2014; Pentzold et al., 2015). For instance, some glucosinolate-adapted *Pieris* species are metabolically adapted to HCN (Stauber et al., 2012) while alliarinoside has been shown to target other *Pieris* species (Renwick et al., 2001; Davis et al., 2015). Interestingly, *Pieris rapae* larvae may be resistant to both of these glucosinolate-derived alternative defense types (Stauber et al., 2012; Frisch et al., 2014). The low amounts and slow release of HCN detected in the present study makes it unlikely that glucosinolate-derived HCN in disrupted *A. petiolata* leaf tissue has significant allelopathic or insect deterring effects. However, the HCN response could be higher in other parts, such as roots or floral parts high in indole glucosinolates, and target yet unknown antagonists.

### Evolution of hydroxynitrile glucoside biosynthesis from the glucosinolate pathway

The known hydroxynitrile glucosides are derived from valine, leucine, isoleucine, phenylalanine, tyrosine, and in specific plants the non-protein amino acid pentenyl glycine (Lechtenberg and Nahrstedt, 1999; Bjarnholt and Møller, 2008). In this study we demonstrate that alliarinoside (14) is biosynthesized from methionine (1) (Figure 6) and hence is the only known hydroxynitrile glucoside derived from this amino acid. Additionally, the observed production of the alliarinoside aglucon (12) from sinigrin (15) (Figure 6) suggests that alliarinoside biosynthesis has evolved from the biosynthesis and metabolism of glucosinolates. If so, the alliarinoside pathway thus differs radically from the hydroxynitrile glucoside pathways in *Sorghum bicolor* (Poales), *Manihot esculenta* (Malpighiales) and *Lotus japonicus* (Fabales) in which the amino acid-derived oxime is the substrate for multifunctional P450s and possibly other enzymes forming the hydroxynitrile that is glucosylated into the hydroxynitrile glucoside (Takos et al., 2011; Frisch and Møller, 2012; Saito et al., 2012). Amino acid-derived oximes are common intermediates in different biosynthetic pathways for glucosinolates as well as cyanogenic and non-cyanogenic hydroxynitrile glucosides (Halkier and Gershenzon, 2006; Gleadow and Møller, 2014). The oxime producing enzyme is in all cases a CYP79, and it has always been a mystery why the compounds co-occur in so few species. In glucosinolate biosynthesis the oxime metabolizing enzyme is a CYP83 producing an aci-nitro compound, whereas in hydroxynitrile glucoside biosynthesis the second enzyme is a multifunctional cytochrome P450 enzyme, in most cases known a CYP71, catalyzing a dehydration of the oxime to form a nitrile, followed by a hydroxylation to a hydroxynitrile. It has now been shown that CYP71E1 from the sorghum plant initially converts the (*E*)-oxime produced by CYP79A1 into the corresponding (*Z*)-isomer, and it is known that the dehydration to form a nitrile is only possible for this isomer of the oxime (Clausen et al., 2015). On the other hand, it was demonstrated that the CYP83 enzyme strictly utilizes the (*E*)-oxime as substrate, and in both cases the oximes are bound to the enzyme in the specific configuration required for the catalytic reaction (Clausen et al., 2015). This means that a multifunctional CYP83 catalyzing both production of nitriles and aci-nitro compounds cannot exist. It then follows that glucosinolates and hydroxynitrile glucosides can only co-exist if the plant harbors both CYP83 and CYP71E1 type enzymes, or if one of the pathways is different from the traditional ones. Our results suggest that the biosynthesis of alliarinoside and sinigrin in *A. petiolata* does not bifurcate at the level of the homomethionine-derived oxime (3) (Figure 2), meaning that CYP83 and CYP71E1 type enzymes do not co-exist in this plant. With sinigrin being a likely precursor for alliarinoside it appears that this hydroxynitrile glucoside pathway results from convergent evolution, hereby providing new insights on independent evolution of specialized metabolites.

The mechanisms and intermediates in the pathway converting sinigrin to the alliarinoside aglucon, (*Z*)-4-hydroxy-2-butenenitrile, are yet elusive. The formation of (*Z*)-4-hydroxy-2-butenenitrile was observed under experimental conditions simultaneously resulting in myrosinase-dependent formation of well-known sinigrin degradation products. Accordingly, (*Z*)-4-hydroxy-2-butenenitrile production most likely also involves sinigrin hydrolysis.

If the observation of the alliarinoside aglycone being derived from sinigrin degradation in homogenates is relevant for alliarinoside biosynthesis in intact plants, it would be in accordance with other recent reports of glucosinolate metabolism in living tissues (Agerbirk and Olsen, 2012). Glucosinolate hydrolysis in intact tissues of *A. thaliana* mediates innate immune responses to pathogens (Bednarek et al., 2009; Clay et al., 2009; Møller, 2010). Further evidence of glucosinolate turnover in the Brassicaceae comes from radiolabelled investigations (Svanem et al., 1997) and studies of changes in the glucosinolate pool during development (Petersen et al., 2002; Brown et al., 2003; Falk et al., 2007). Glucosinolate turnover in living tissues with no pathogen challenge has been suggested as a mechanism to mobilize the sulphur and nitrogen of glucosinolates for primary metabolism during nutrient limitation (Falk et al., 2007; Piotrowski, 2008), but this proposal calls for conclusive evidence. The molar ratio of sinigrin to alliarinoside was 1:3 in leaves so the metabolism of sinigrin into large amounts of alliarinoside (0.4% FW) would indicate a profound formation and turnover of glucosinolates in *A. petiolata*. In the model plant *A. thaliana* a large proportion of glucosinolates are stored in socalled S-cells (sulfur rich cells). These cells were also shown to contain myrosinase proteins (Koroleva et al., 2010; Koroleva and Cramer, 2011) and ESP (Burow et al., 2007) (*A. thaliana* does not contain TFP). If *A. petiolata* harbors a similar organization the putative enzymes converting sinigrin to alliarinoside following myrosinase/TFP mediated degradation may be found in S-cells. In future investigations we will aim at identifying the cell- and tissue specific distribution of sinigrin and alliarinoside in *A. petiolata* using mass spectrometry based imaging (MSI) techniques (Li et al., 2013, 2014; Bjarnholt et al., 2014). Genomic resources are not available for *A. petiolata* and hence the further exploration of alliarinoside biosynthesis still relies on biochemical characterizations. A first step would be to produce radiolabelled sinigrin to be administered to *A. petiolata* leaves disclosing if sinigrin is the precursor of alliarinoside *in planta*.

A broad glucosinolate degradation product profile was detected in *A. petiolata* leaf homogenate. The semi-quantitative detection of sinigrin-derived thiocyanate and epithionitrile in addition to isothiocyanate was in agreement with the recent identification of a thiocyanate-forming protein, ApTFP1, expressed in *A. petiolata* cotyledons (Kuchernig et al., 2012). Furthermore, the sinigrin-derived simple nitrile, 3-butenenitrile, was detected semi-quantitatively in the present study. Though small amounts of simple nitriles may form spontaneously upon glucosinolate hydrolysis (Burow et al., 2006), the demonstrated presence of 3-butenenitrile may indicate presence of an unidentified specifier protein in addition to ApTFP1, which was found not to promote 3-butenenitrile formation from sinigrin degradation *in vitro* (Kuchernig et al., 2012).

## Concluding remarks

Collectively, we have shown that sinigrin metabolism in *A. petiolata* is remarkably varied, as summarized in Figure 6. *A. petiolata* has apparently evolved the ability to diversify the formation of glucosinolate-derived products into the formation of alliarinoside, a hydroxynitrile glucoside with deterring or toxic properties against glucosinolate-adapted specialists (Haribal et al., 2001; Renwick et al., 2001; Davis et al., 2015). Furthermore, *A. petiolata* has a remarkably broad profile of glucosinolate-derived products in disrupted tissues including sinigrin-derived isothiocyanate, epithionitrile, simple nitrile, and thiocyanate as well as the observed thiocyanate-derived HCN release. This diversity in glucosinolate degradation products as well as the combined formation of glucosinolates and hydroxynitrile glucosides highlight that glucosinolate metabolism in intact plants is highly complex and often species-dependent. The production of the rare defense compound alliarinoside is commonly viewed as an example of a “second line of defense” in a crucifer (Feeny, 1977; Renwick, 2002). Such second lines of defenses are hypothesized to have arisen during an evolutionary arms race between plants and adapting enemies. Biosynthesis of HCN and apparently also alliarinoside from glucosinolates is particularly remarkable if derived from unusual glucosinolate hydrolysis products (allyl thiocyanate and 3,4-epithiobutenenitrile), as that would in fact be a “third line of defense” depending on three consecutive innovations: glucosinolates, thiocyanate forming protein, and the yet elusive biosynthesis from thiocyanates to HCN and possibly epithionitrile to alliarinoside.

## Experimental procedures

### Plant material and growth conditions

Rosette leaves of *A. petiolata* were harvested from first year plants taken from the field in August and September 2010–2012 at three Danish locations (Køge, Frederiksberg, and Valby) and grown in the greenhouse. *Brassica juncea* plants were from seeds collected in the University Gardens, Frederiksberg Campus, University of Copenhagen. Seeds of *Arabidopsis thaliana* ecotype Col-0 were obtained from Lehle Seeds, Round Rock, Texas, USA. *Lotus japonicus* cv. MG-20 plants were provided by Adam Takos from our laboratory. For each experiment, a relatively constant blend of leaves from different plants was used in an attempt to optimize the chance of getting biosynthetically active tissues and minimize variation between experiments. All plants were grown in soil in a greenhouse with 16 h light and minimum day/night temperatures of 18/15°C (Frisch et al., 2014).

### Chemicals

All chemicals and reagents were obtained from http://www.sigmaaldrich.com with the following exceptions: Linamarin (AG Scientific, San Diego, California, USA www.sigmaaldrich.com with the following exceptions: Linamarin (AG Scientific, San Diego, California, USA), 3-butenamide and 4-(methylthio)butanenitrile (Santa Cruz Biotechnology, Santa Cruz, California, USA), [UL-^14^C]-L-aspartate (Larodan Fine Chemicals, Malmö, Sweden). L-Homomethionine and [UL-^14^C]-methionine were generously provided by Professor Jonathan Gershenzon, Max Planck Institute for Chemical Ecology, Jena, Germany.

### Chemical synthesis

**Figure 9:**
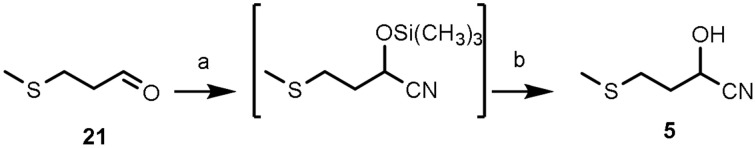
**Chemical synthesis of 2-hydroxy-4-(methylthio)butanenitrile (5)**. *Reagents and conditions*: (a) Trimethylsilyl cyanide [TMSCN: (CH_3_)_3_SiCN], solid lithium perchlorate (LiClO_4_), room temperature (r.t.), 2 h, quantitative; (b) THF, HCl(3 M), 65°C, 1 h, 84% overall yield.

Shortly, chemical synthesis was performed as follows (details in Methods S1). Commercially available 3-(methylthio)propionaldehyde was converted in a one-pot reaction step into its cyanohydrin, 2-hydroxy-4-(methylthio)butanenitrile (5), in an overall yield of 84% by treatment with trimethylsilyl cyanide (TMSCN)-lithium perchlorate (Azizi and Saidi, 2003) and subsequent acid treatment (Gassman and Talley, 2003) (Figure 9).

**Figure 10:**
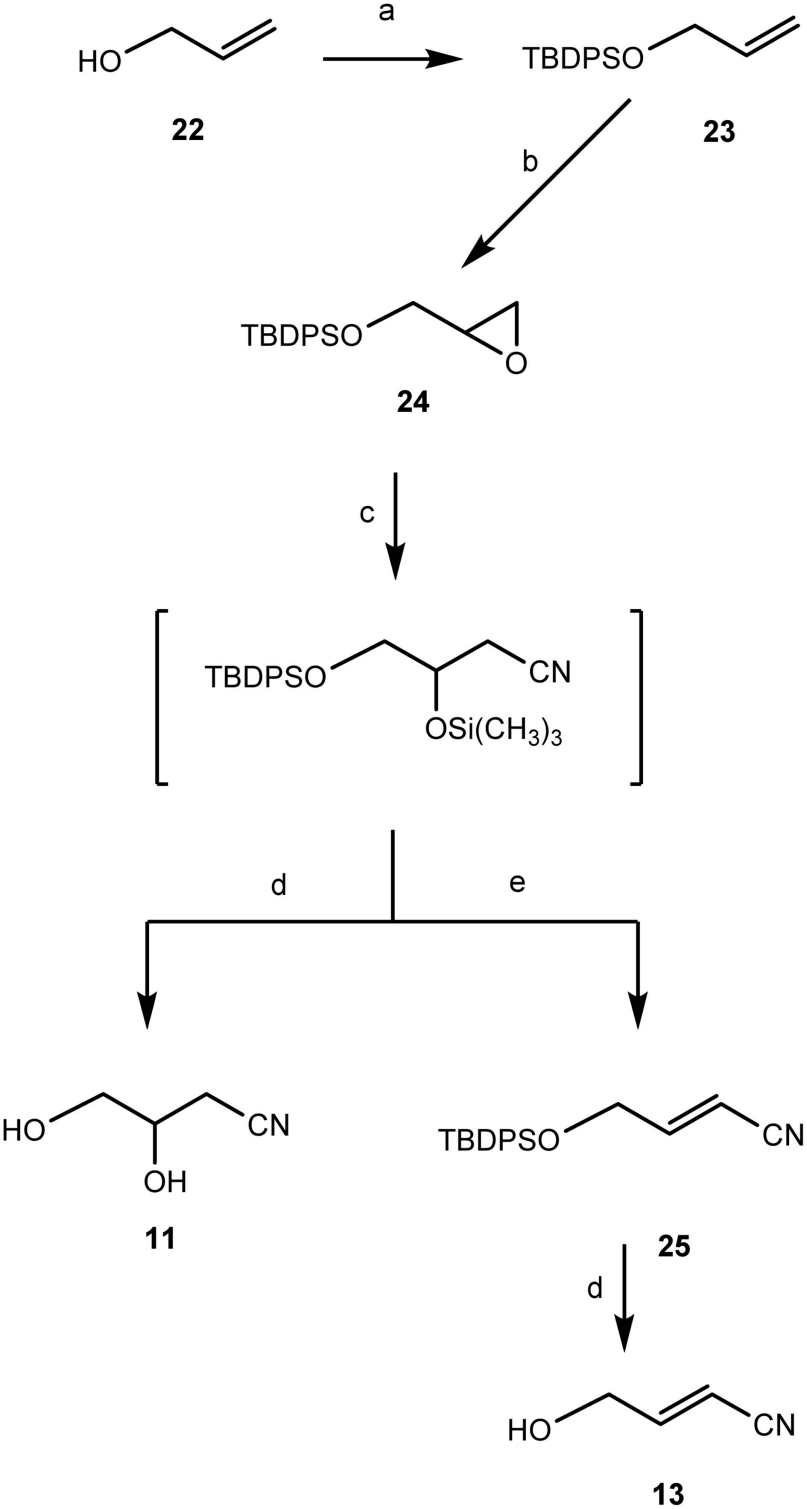
**Chemical synthesis of 3,4-dihydroxybutanenitrile (11) and (***E***)-4-hydroxy-2-butenenitrile (13)**. *Reagents and conditions*: (a) TEA, DMAP, TBDPSiCl, CH_2_Cl_2_, r.t., 1 h, 97%; (b) *m*-CPBA, ClCH_2_CH_2_Cl, 85°C, 30 min, 95%; (c) Trimethylsilyl cyanide [TMSCN: (CH_3_)_3_SiCN], solid lithium perchlorate (LiClO_4_), 85°C, 1 h; (d) TBAF/THF, r.t. 1 h; (e) Py/POCl_3_, 85°C, 2 h, 87% yield.

3,4-Dihydroxybutanenitrile (11) and (*E*)-4-hydroxy-2-butenenitrile (13) were chemically synthesized from allyl alcohol 22 as shown in Figure 10. In this synthetic scheme, the OH-group of 22 was protected with a *tert*-butyldiphenylsilyl group *via* silylation with *tert*-butyldiphenylchlorosilane using the DMAP-TEA method (Chaudhary and Hernandez, 1979) to give 23 quantitatively. Epoxidation of 23 with *m*-chloroperbenzoic acid in C_2_H_2_Cl_2_ under reflux for 30 min afforded *O*-tert-butyldiphenylsilyl-protected-(R,S)-2,3-epoxypropyl alcohol (24) quantitatively. Ring-opening of epoxide 24 using TMSCN under solvent free conditions (Mirmashhori et al., 2006) and subsequent treatment of the formed cyanosilylated product with tetra-*n*-butylammonium fluoride (TBAF) in tetrahydrofuran (THF) afforded the dihydroxynitrile 11 in excellent yield. In a separate one-pot reaction approach, the ring-opening of epoxide 24 was followed by treatment of the formed trimethylsilyloxy nitrile with pyridine and phosphoryl chloride (Oda et al., 1979) to afford an isomeric mixture of (*E*)- and (*Z*)-isomers in the ratio of *E*/*Z* = 2:1 of the O-*tert*-butyl-diphenylsilylchloride- (TBDPS-) protected unsaturated nitriles (25). De-protection of 25 afforded the pure *(E)*-isomer (13) in 79% yield. It was not possible to obtain the *(Z)*-isomer.

All the synthesized compounds were characterized using electrospray ionization mass spectrometry and ^1^H and ^13^C-NMR to verify the structural assignment and purity by comparison to literature data. (*Z*)- and (*E*)-alliarinoside were prepared as described elsewhere (Olsen et al., 2014).

### [UL-^14^C]-methionine administration to leaves and formation of ^14^c-labeled alliarinoside

[UL-^14^C]-Methionine (37 KBq, specific activity: 10.175 TBq mol^−1^) was administered through the petiole of the youngest fully unfolded rosette leaf (*n* = 4; approximately 40 mg FW leaf^−1^) of *A. petiolata* plants. After uptake, additional water was provided and the leaves were incubated for 24 h (100 μmol photons m^−2^ s^−1^, 21°C and 70% relative humidity). Metabolites were extracted by boiling in 85% MeOH (500 μL, 3 min). The supernatant was evaporated to dryness and the residue was re-dissolved in 50 μl H_2_O. Following extraction in EtOAc (1 volume, 3 times), the aqueous phase was fortified with 0.1 mM KNO_3_ to provide a surplus of defined glucosinolate counter ions and accordingly similar migration of endogenous sinigrin and the sinigrin potassium salt used as standard. Aliquots were applied to silica gel TLC plates (type 60 F_254_, Merck) and dried. To promote uniform migration, the TLC plates were initially developed in MeOH to 2 cm above origin and dried. This focusing procedure was performed 5 times. Subsequently, the plates were fully developed in either of two solvent systems for separation of hydroxynitrile glucosides and glucosinolates, respectively: EtOAc: Me_2_CO: CH_2_Cl_2_: MeOH: H_2_O [20:15:6:5:4 (v/v)] or 2-propanol: EtOAc: H_2_O [7:1:1 (v/v)]. Radiolabelled compounds were visualized by Storage Phosphor Screens (Amersham Biosciences) and a Typhoon Trio scanner (GE Healthcare). Unlabeled standards were visualized by charring with 10% H_2_SO_4_ in MeOH.

### Preparation of leaf homogenates, microsomes, and soluble protein extracts

For all types of samples leaf homogenates were prepared by grinding young rosette leaves from minimum three *A. petiolata* plants in liquid nitrogen using mortar and pestle. Microsomes and soluble proteins were isolated at 4°C. *A. petiolata* leaves (10 g) were ground with 1 g polyvinylpolypyrrolidone and 20 ml isolation buffer [250 mM KP_i_ (pH 8.0), 250 mM sucrose, 100 mM ascorbic acid, 50 mM NaCl, 2 mM EDTA, 2 mM DTT and 1 mM phenylmethylsulfonyl fluoride (PMSF)]. The homogenate was filtered through two layers of 22 μm nylon mesh and the filtrate was centrifuged (10 min, 27,000 × g) to remove cell debris. Ultracentrifugation (1 h, 194,000 × g) of the supernatant pelleted the microsomes, which were resuspended in isolation buffer in a Potter-Elvehjem homogenizer and dialyzed (1 h, Spectra/Por dialysis tubing, 6000–8000 Da MW cut off) against dialysis buffer (50 mM KP_i_ (pH 8.0), 2 mM DTT, 100 mM ascorbic acid) and twice against dialysis buffer devoid of ascorbic acid (1 h and overnight). Aliquots were frozen in liquid N_2_ and stored at −80°C.

The soluble proteins obtained in the supernatant from the ultracentrifugation step were desalted twice in dialysis buffer devoid of ascorbic acid using PD-10 columns (5000 Da MW cut off, GE Healthcare). The soluble protein fraction was concentrated using Amicon Ultra Centrifugal filters (10,000 mW cut off; Millipore), frozen in liquid nitrogen and stored at −80°C.

### Enzyme activity investigations

Enzyme activities in the leaf homogenate, isolated microsomes or the soluble protein fraction were monitored (total volume: 100 μL) in 50 mM KP_i_ (pH 8.0) and 2 mM DTT plus leaf homogenate (30 mg FW), microsomes (from 375 to 625 mg FW) or soluble proteins (from 33 to 120 mg FW). All putative substrates were tested at 1 mM final concentration except sinigrin which was used at 10 mM. When relevant, samples were supplemented with the following cofactors: 2 mM NADPH, 10 mM 2-oxoglutarate, 10 mM ascorbate, 1 mg mL^−1^ catalase from bovine liver. Samples prepared to investigate the effect of Fe^2+^ on 2-ODD activity or nitrile formation during glucosinolate degradation were fortified with 100 μM FeSO_4_. Samples were incubated at r.t. and 250 r.p.m. for 30 min (microsomes) or 2 h (homogenate or soluble proteins) followed by extraction for GC-MS. Mixing of buffer with leaf homogenate, microsomes, or soluble protein took place under ice cold conditions, and the control samples were extracted immediately after, i.e., at time point zero.

### GC-MS analyses and sample preparation

Aqueous samples (100 μL) were acidified by 1 M HCl (5 μL), supplemented with benzonitrile as internal standard (final concentration: 100 ng μL^−1^) and extracted with CH_2_Cl_2_ (3 times, 4 volumes). The combined CH_2_Cl_2_ phase was dried over MgSO_4_, filtered through glass wool in a Pasteur pipette and concentrated to approximately 10 μL under a N_2_ atmosphere. The sample was diluted with CHCl_3_ (total volume: 100 μL), TMSCN-derivatized (50 μL sample, 20 μL TMSCN) (Khakimov et al., 2013) and analyzed by direct injection in the GC-MS. To correct for variation in sample preparation and injection volume, the internal standard EIC peak area was used to calculate the relative peak area of other analyses.

GC-MS was performed in a Turbomass spectrometer coupled to an Autosystem XL gas chromatograph (both from PerkinElmer, USA). The transfer line was kept at 200°C. Split injection (1:10) of 1 μL at 200°C, He as carrier gas (1 mL/min). Chromatographic separation was obtained using an SGE column (SGE 30QC2/BPX5-0.25, P/N 054142, 30 m × 0.22 mm, 0.25 μm film thickness). The oven temperature program was as follows: 80°C for 2 min, 80–160°C at 5°C min^−1^, 160–280°C at 20°C min^−1^, 280°C for 3 min. The ion source was run in EI mode (70 eV) at 200°C. The mass range *m*/*z* 50–500 was acquired.

SPME-GC-MS analyses were performed using the same instrument fitted with the same column. Load time 1 min at 250°C. The oven temperature program was as follows: 40°C for 2 min, 40–250°C at 10°C min^−1^, 250°C for 2 min. The mass range *m*/*z* 50–300 was acquired. Manual SPME sampling was carried out by exposing a Carboxen fiber (Supelco 57334-U, “Light Blue”) to the head space from 100 μL aqueous stirred sample at the bottom of a 1.5 mL septum sealed vial (HPLC vial). The exposure was maintained for 20 min at 80°C.

Compounds were identified by known standards, except when mentioned otherwise in text and figure legends.

### Glucoside extraction and LC-MS analyses

For extraction of glucosides, leaf material was homogenized in liquid N_2_, and frozen aliquots (5–10 mg) were boiled in 85% MeOH (300 μL, 3 min), cooled on ice and diluted 5 times in H_2_O. Linamarin was added as internal standard (final concentration 40 μM) and samples were filtered through a 0.45 μm MultiScreen HTS HV filterplate (Millipore) before LC-MS analysis.

LC-MS analysis was carried out using an Agilent 1100 Series LC (Agilent Technologies, Germany) coupled to a Bruker HCT-Ultra ion trap mass spectrometer (Bruker Daltonics, Bremen, Germany). For standard glucoside analyses, separation was carried out as described (Pičmanová et al., 2015) using a Zorbax SB-C18 column (Agilent; particle size: 1.8 μm, 2.1 × 50 mm, flow rate: 0.2 mL min^−1^) and mobile phases as follows: A, water with 0.1% (v/v) HCOOH and 50 μM NaCl; B, acetonitrile with 0.1% (v/v) HCOOH. Analysis and quantification of the very polar alliarinoside and sinigrin was likewise carried out as described (Frisch et al., 2014), with the same mobile phases as above, but the column exchanged for a Luna C8(2) column (Phenomenex; particle size: 3 μm, 100 A, 150 × 2.0 mm) preceded by a Gemini C18 SecurityGuard (Phenomenex; 4 × 2 mm). Glucosides were detected as sodium adducts and their identities were confirmed by comparison to authentic standards. Accurate masses were determined on a Bruker microTOF-Q spectrometer using LC parameters similar to those in the “standard” analysis above.

### HCN detection

The ability of *A. petiolata* and specific metabolites to release HCN was studied using assay mixtures (total volume: 200 μL) prepared on ice in 1.5 mL microcentrifuge tubes containing ice cold 50 mM MES-buffer (pH 6.0) and 30 mg leaf homogenate, which was obtained by grinding young fully unfolded leaves of three or more individuals in liquid N_2_ using a mortar and pestle. Individual samples were spiked with alliarinoside (final concentration: 8 nmol mg FW^−1^) or other compounds (final concentration: 10 nmol mg FW^−1^). Myrosinase (from *Sinapis alba*, Sigma-Aldrich) was included (final concentration: 100 μU μL^−1^) in samples investigating *A. petiolata*-independent HCN release from sinigrin. To trap released HCN, an alkaline trap consisting of a PCR tube containing 240 μL 1 M NaOH was positioned in each microcentrifuge tube. The samples were incubated (28°C, 500 r.p.m., 18 h) and frozen in liquid N_2_ before the alkaline trap was removed. To stop diffusion from the leaf suspension, 40 μL 6 M NaOH was added to the microcentrifuge tube. The König reaction modified by Lambert et al. (König, 1904; Lambert et al., 1975) was used to quantify the amount of HCN in the alkaline trap and to assess the presence of non-diffusible reactants in the leaf suspension that also provided a color reaction. The samples (60 μL aliquot or an appropriate dilution) were analyzed (technical duplicates) in a 96-well microtiter plate by sequential rapid addition of the following reagents: 12.5 μL glacial HOAc, 50 μL N-chlorosuccinimide (1 g l^−1^) combined with N-succinimide (2.5 g L^−1^), and finally 50 μL barbituric acid (60 g L^−1^) in 30% pyridine (v/v). After incubation (10 min, r.t.), the absorbance (A) was determined in a SpectraMax M5 microplate reader (Molecular Devices). The HCN response (A_585_–A_750nm_) was quantified relative to a standard curve of KCN in 1 M NaOH.

For HCN detection by the less sensitive Feigl-Anger paper (Takos et al., 2010), assay mixtures were prepared as described above except that the amount of *A. petiolata* homogenate was increased to 60 mg FW sample^−1^ and the alkaline trap volume was decreased to 60 μL 1 M NaOH. In a 96-well microtiter plate, 60 μL alkaline trap solution was added 10 μL 4 M H_2_SO_4_. The wells were immediately covered with Feigl-Anger paper and the plate lid. Following incubation (1 h, r.t., 250 r.p.m.), color development over individual wells was documented by scanning. A positive control was prepared containing 4 mg FW of homogenized trefoils from *Lotus japonicus* MG20.

### Conflict of interest statement

The authors declare that the research was conducted in the absence of any commercial or financial relationships that could be construed as a potential conflict of interest.
