# Supplementary material for: Diversified glucosinolate metabolism: biosynthesis of hydrogen cyanide and of the hydroxynitrile glucoside alliarinoside in relation to sinigrin metabolism in Alliaria petiolata
Source: Front Plant Sci. 2015 Oct 31;6:926. doi: 10.3389/fpls.2015.00926 (PMC4628127; doi:10.3389/fpls.2015.00926)
Supplement: Supplementary file 1 [file Image1.PDF]

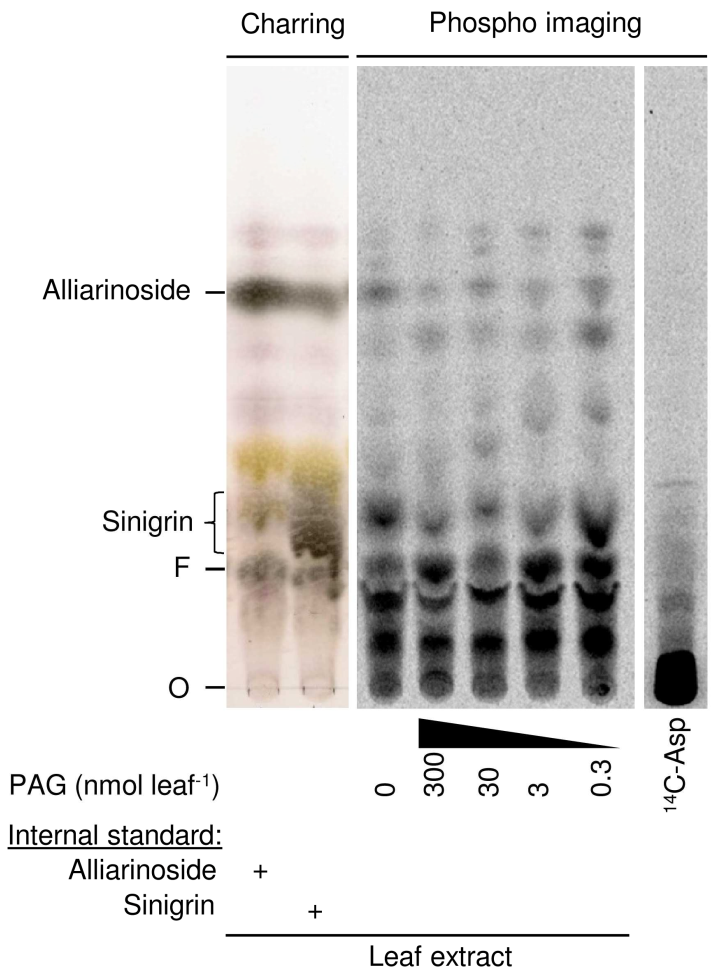

Figure S1: An alternative approach to investigation of methionine (1) as a precursor of alliarinoside (14).

Methionine with a radiolabel positioned at the methyl group or at the sulphur atom is readily available from commercial sources. However, this carbon atom as well as the sulphur atom of the amino acid is not retained during metabolism of methionine into sinigrin. Likewise, our originally suggested biosynthetic route of alliarinoside from methionine excluded the use of these atoms from the amino acid precursor as tracers. As [UL-^{14}C]-methionine is much more difficult to obtain, we took a different approach to investigate methionine as a precursor of alliarinoside. Higher plants *de novo* synthesize methionine via three convergent pathways. The carbon backbone originates from aspartate, whereas the sulphur atom and the methyl group are derived from cysteine and serine, respectively (Ravanel et al., 1998). [UL-^{14}C]-Aspartate is commercially available and can thus be used as precursor for methionine ^{14}C-labelled in C_1-C_4.

However, apart from methionine aspartate is also metabolized to asparagine, threonine, lysine and isoleucine (Azevedo et al., 2006). The route from aspartate to methionine can be blocked specifically by propargyl glycine (PAG), which acts as a suicide, active site-directed, inhibitor of the enzyme cystathionine γ-synthase catalysing the first committed step in the methionine pathway (Thompson et al., 1982; Ravanel et al., 1998). Hence, initial administration of PAG to leaves of *A. petiolata* and subsequent administration of [UL-^{14}C]-aspartate allowed us to investigate if the radiolabelled amino acid was incorporated into alliarinoside via the methionine pathway. The leaf feeding assay was performed as described for [UL-^{14}C]-methionine feeding with the modifications that the *A. petiolata* leaves were fed 0.3-300 nmol DL-PAG in 4 µl aqueous solution or the same volume of H_2O prior to addition of [UL-^{14}C]-L-aspartate (38.5 KBq leaf^{-1}; 7.696 TBq mol^{-1}) instead of [UL-^{14}C]-methionine. The figure shows results of a representative feeding experiment analysed by TLC (EtOAc: Me_2CO: CH_2Cl_2: MeOH: H_2O (20:15:6:5:4 (v/v))) and subsequently visualized by phosphor imaging and charring of unlabelled compounds, including glucosides. O: origin; F: methanol focus line.

^{14}C-alliarinoside and ^{14}C-sinigrin were present in all leaves fed ^{14}C-aspartate. Furthermore, it is apparent that administration of increasing amounts of PAG decreased production of ^{14}C-alliarinoside and ^{14}C-sinigrin. Biological differences in biosynthetic rate among individual leaves and uneven uptake and distribution of PAG may explain that this tendency was not more outspoken. Collectively, these results showed that [UL-^{14}C]-aspartate was metabolized into ^{14}C-alliarinoside via the methionine pathway. Thus, methionine is the amino acid precursor of alliarinoside.

Efforts to detect alliarinoside biosynthesis using administration of [UL-^{13}C], ^{15}N-L-methionine and subsequent LC-MS analysis were unsuccessful. Most likely, a low biosynthetic yield resulting in a minor chromatographic peak of ^{13}C, ^{15}N-alliarinoside was masked by the large amount of endogenous, unlabelled alliarinoside already present in the leaf. Hence, more sensitive radiolabelled approaches were pursued as described.

References

Azevedo, R.A., Lancien, M. and Lea, P.J. (2006). The aspartic acid metabolic pathway, an exciting and essential pathway in plants. *Amino Acids*, 30, 143-162.

Ravanel, S., Gakiere, B., Job, D. and Douce, R. (1998). The specific features of methionine biosynthesis and metabolism in plants. *PNAS* 95, 7805-7812.

Thompson, G.A., Datko, A.H. and Mudd, S.H. (1982). Methionine Synthesis in Lemna: Inhibition of Cystathionine γ-Synthase by Propargylglycine. *Plant Physiol.*, 70, 1347-1352
