# Supplementary material for: Diversified glucosinolate metabolism: biosynthesis of hydrogen cyanide and of the hydroxynitrile glucoside alliarinoside in relation to sinigrin metabolism in Alliaria petiolata
Source: Front Plant Sci. 2015 Oct 31;6:926. doi: 10.3389/fpls.2015.00926 (PMC4628127; doi:10.3389/fpls.2015.00926)
Supplement: Supplementary file 2 [file Image2.PDF]

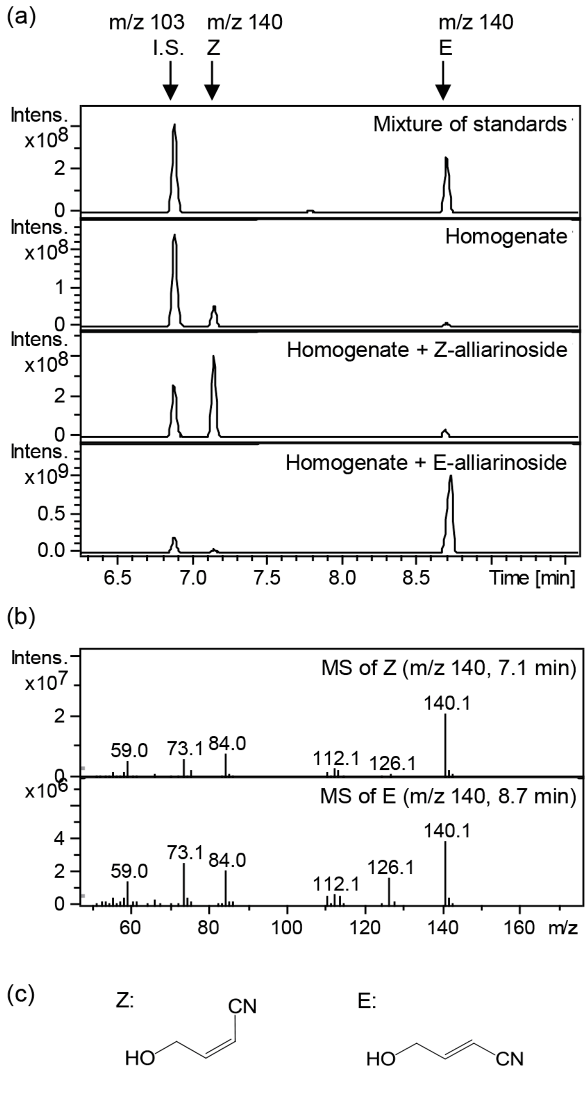
(a)
m/z 103
I.S.
m/z 140
Z
m/z 140
E
Intens.
x10^8
Mixture of standards
Homogenate
Homogenate + Z-alliarinoside
x10^9
Homogenate + E-alliarinoside
Time [min]
(b)
x10^7
MS of Z (m/z 140, 7.1 min)
x10^6
MS of E (m/z 140, 8.7 min)
59.0
73.1
84.0
112.1
126.1
140.1
m/z
(c)
Z:
E:
HO
CN

Figure S2: Identification of (*Z*)-4-hydroxy-2-butenenitrile (12) in leaf homogenate. Chemical synthesis of a standard of the alliarinoside aglucon, (*Z*)-4-hydroxy-2-butenenitrile (**12**), was challenging due to chemical isomerization to the (E)-isomer (**13**). Thus, another approach was taken to confirm the presence of (*Z*)-4-hydroxy-2-butenenitrile in leaf homogenate. (Panel a) The GC-MS chromatograms show EIC(140;103). Benzonitrile (*m*/*z* 103) was included in all samples as internal standard (I.S.). The standard of the (*E*)-isomer (E) was detected as the trimethylsilyl- (TMS-) derivative (*m*/*z* 140) at a retention time (RT) of 8.7 min and was found present in leaf homogenate. Another homogenate compound (*m*/*z* 140, 7.1 min) had a very similar mass spectrum (panel b) and was thus suspected to be (*Z*)-4-hydroxy-2-butenenitrile (Z). To confirm this, leaf homogenate was spiked with chemically synthetized (*Z*)-alliarinoside or (*E*)-alliarinoside followed by incubation to allow endogenous enzyme activity. The resulting metabolites were analysed by GC-MS. Samples spiked with (*E*)-alliarinoside contained more (*E*)-4-hydroxy-2-butenenitrile than untreated samples as expected from hydrolysis of the glucoside to the corresponding aglucon. In samples spiked with (*Z*)-alliarinoside a markedly increase in the compound at 7.1 min was seen, hereby confirming the identity as (*Z*)-4-hydroxy-2-butenenitrile.
