# Supplementary material for: Diversified glucosinolate metabolism: biosynthesis of hydrogen cyanide and of the hydroxynitrile glucoside alliarinoside in relation to sinigrin metabolism in Alliaria petiolata
Source: Front Plant Sci. 2015 Oct 31;6:926. doi: 10.3389/fpls.2015.00926 (PMC4628127; doi:10.3389/fpls.2015.00926)
Supplement: Supplementary file 3 [file Image3.PDF]

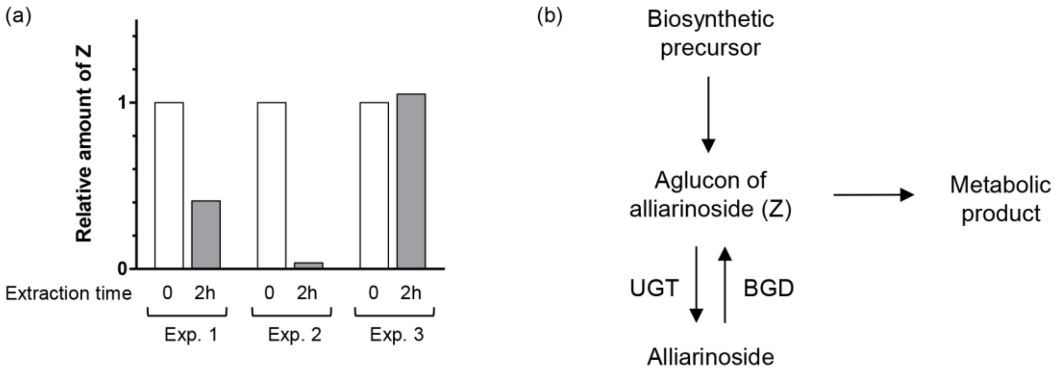

Figure S3: Variation in the detected amount of alliarinoside aglucon (Z; (*Z*)-4-hydroxy-2-butenenitrile (12)) in *A. petiolata* leaf homogenates.

a) The relative amount of alliarinoside aglucon in individually analysed *A. petiolata* homogenates at time point zero (defined as reference = 1) and after 2 h incubation to allow enzyme catalysed activities. Data acquired by GC-MS from three experiments (Exp. 1 – Exp. 3) with biologically different material are shown (mean of technical replicates: n=2 except Exp. 1: n=1). There was a tendency to decreased amounts of alliarinoside aglucon after incubation.

b) Factors influencing the detected amount of alliarinoside aglucon at a given time. UGT, UDP-glucosyl transferase; BGD, β-glucosidase.
