# Supplementary material for: Diversified glucosinolate metabolism: biosynthesis of hydrogen cyanide and of the hydroxynitrile glucoside alliarinoside in relation to sinigrin metabolism in Alliaria petiolata
Source: Front Plant Sci. 2015 Oct 31;6:926. doi: 10.3389/fpls.2015.00926 (PMC4628127; doi:10.3389/fpls.2015.00926)
Supplement: Supplementary file 4 [file Image4.PDF]

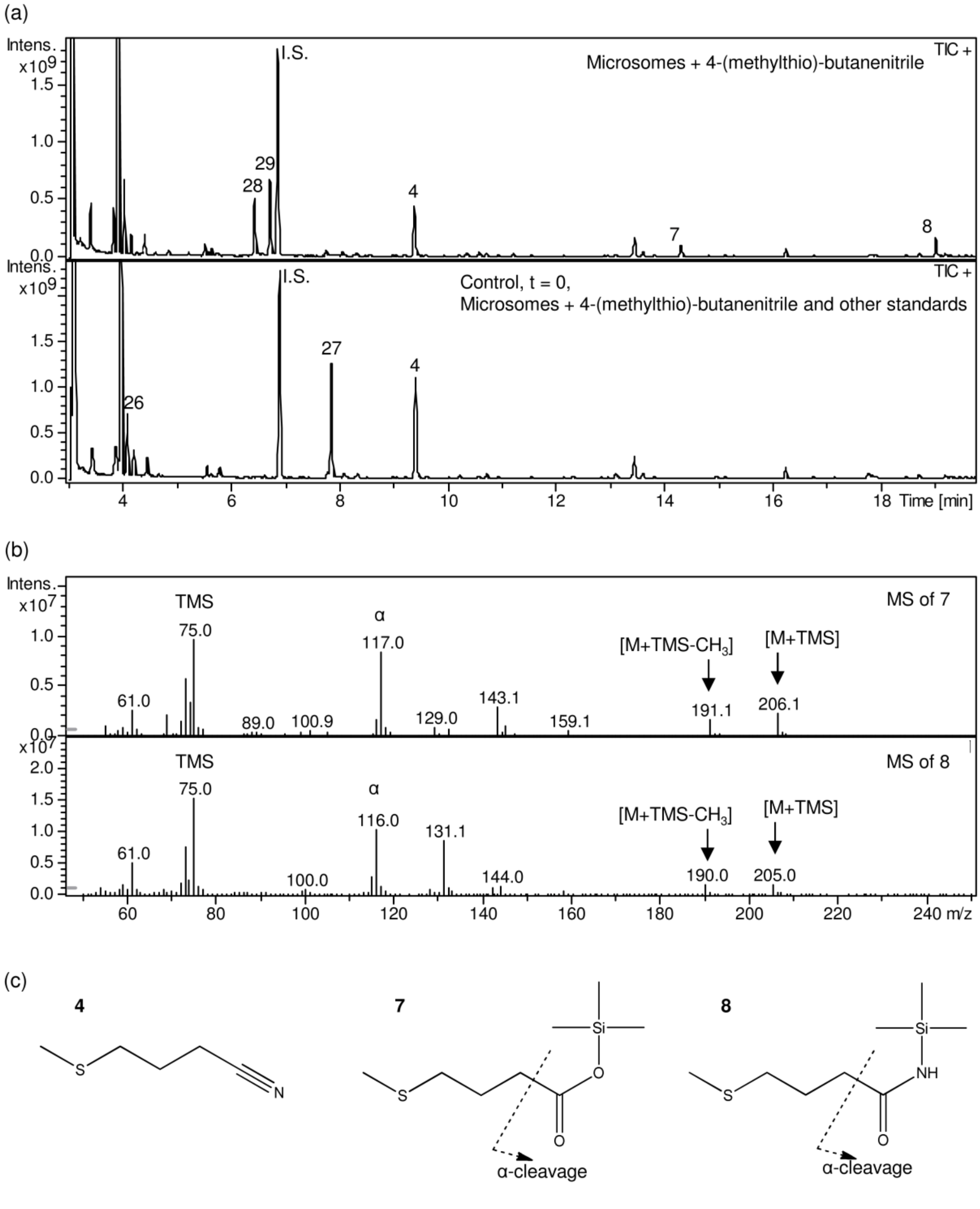

Figure S4: 4-(Methylthio)butanenitrile (4) was metabolized to the corresponding acid (7) and amide (8), which indicates nitrilase and nitrile hydratase activity in *A. petiolata*.

(a) GC-MS total ion chromatograms (TIC) of microsome activity assay samples derivatized with trimethylsilyl cyanide (TMSCN). Compounds **7** and **8** were detected, when microsomes were incubated with 4-(methylthio)butanenitrile (**4**) independently of NADPH supplement (upper panel), but not at time point zero (t=0; lower panel of a).

The same pattern was seen with soluble enzymes instead of microsomes. The lower peak intensity of 4 relative to the internal standard (I.S.) after incubation indicated metabolism of the substrate by the active enzyme fraction. Based on the spectra (b), **7** and **8** were identified as the trimethylsilyl- (TMS-) derivatives of 4-(methylthio)butanoic acid and 4-(methylthio)butanamide, respectively. (c) Chemical structures of the TMS-derivatives showing the major MS fragment from α-cleavage. **26**: 3-butenoic acid standard; **27**: 3-butenamide standard; **28** and **29**: organic solvent impurities; M: molecular ion

These results imply that the acid (**7**) and amide (**8**) were enzyme-formed products from the nitrile (**4**). We suggest that the reactions were catalysed by a nitrilase possessing nitrile hydratase activity (EC 4.2.1.84) in addition to nitrilase activity (EC 3.5.5.1) as observed for NIT1 homologs of other Brassicaceae members (Piotrowski, 2008) as well as nitrilases with specificity for glucosinolate-derived nitriles (Agerbirk et al., 2008). The reactions were observed in both microsomal and soluble enzyme fraction, which may reflect the localization *in vivo* or may result from contamination of the microsomal fraction with cytoplasm as argued for the similar reported differences in cellular localization of Arabidopsis NIT1, NIT2 and NIT3 (Piotrowski, 2008).

Compound **4**, **7** or **8** were not detected in leaf homogenate, microsomes or soluble enzyme fraction, when these were not supplemented with the nitrile. Accordingly, the nitrile (**4**) may be an artificial substrate for *A. petiolata* nitrilases. No other products from 4 were detected including 3-hydroxy-4-(methylthio)butanenitrile (**6**). Hence, we did not obtain evidence supporting the initial hypothesis that 4-(methylthio)butanenitrile (**4**) is the first committed intermediate in the alliarinoside biosynthesis pathway from homomethionine-derived oxime.

References

Agerbirk, N., Warwick, S.I., Hansen, P.R. and Olsen, C.E. (2008). Sinapis phylogeny and evolution of glucosinolates and specific nitrile degrading enzymes. *Phytochemistry*, 69, 2937-2949

Piotrowski, M. (2008). Primary or secondary? Versatile nitrilases in plant metabolism. *Phytochemistry*, 69, 2655-2667
