# Supplementary material for: Diversified glucosinolate metabolism: biosynthesis of hydrogen cyanide and of the hydroxynitrile glucoside alliarinoside in relation to sinigrin metabolism in Alliaria petiolata
Source: Front Plant Sci. 2015 Oct 31;6:926. doi: 10.3389/fpls.2015.00926 (PMC4628127; doi:10.3389/fpls.2015.00926)
Supplement: Supplementary file 5 [file Image5.PDF]

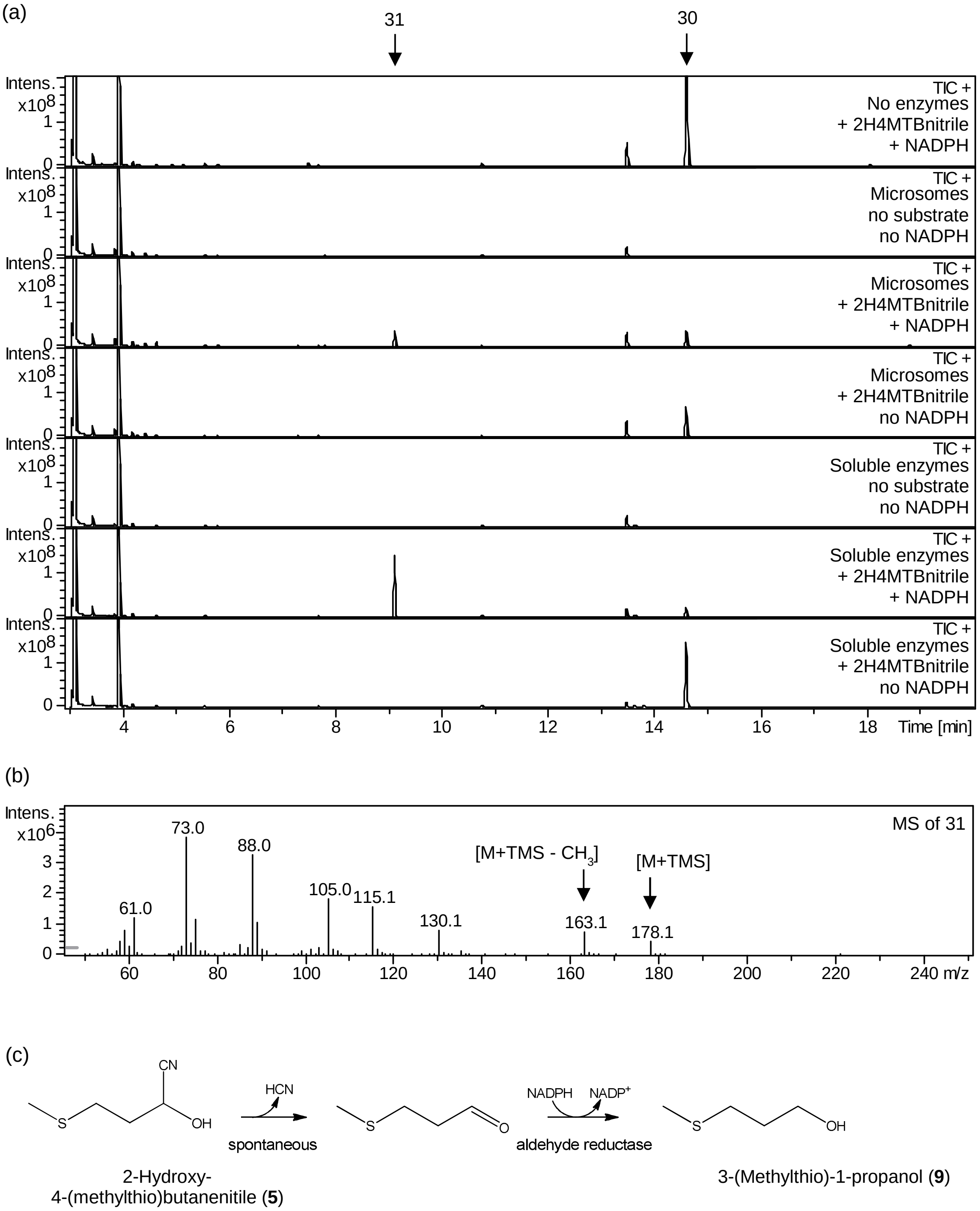

Figure S5: 2-Hydroxy-4-(methylthio)butanenitrile (5) was metabolized to 3-(methylthio)-1-propanol (9).

(a) GC-MS total ion chromatograms (TIC) of enzyme activity assay samples derivatized with trimethylsilyl cyanide (TMSCN). **30** designates the trimethylsilyl- (TMS-) derivative of 2-hydroxy-4-(methylthio)butanenitrile (2H4MTBnitrile) (**5**). Microsomes

as well as soluble enzymes incubated with 2-hydroxy-4-(methylthio)butanenitrile (**5**) produced compound **31** in a NADPH-dependent manner. No other enzymatic products were detected. **31** was identified as the TMS-derivative of 3-(methylthio)-1-propanol (**9**) by comparing the mass spectrum (b) to the library spectrum in the Wiley 9th Edition/ NIST 2011 MS database (ID# 124775). (c) α-Hydroxynitriles such as 2-hydroxy-4-(methylthio)butanenitrile are unstable and spontaneously release HCN, which results in aldehyde formation. It is possible that an NADPH-dependent aldehyde reductase metabolized the derived aldehyde, hereby resulting in the observed production of 3-(methylthio)-1-propanol. Detoxification of reactive aldehydes by NAPDH-dependent reductases is known from monocots and dicots, including Arabidopsis, and some of these cytosolic enzymes have broad substrate specificity (Yamauchi et al., 2011; Gavidia et al., 2002). 2-Hydroxy-4-(methylthio)butanenitrile was not detected in leaf homogenate or enzyme fractions without added substrate. Hence, the observed metabolism may not occur *in planta*. In conclusion, we did not obtain evidence suggesting that 2-hydroxy-4-(methylthio)butanenitrile (**5**) is an intermediate in alliarinoside (**14**) biosynthesis. No internal standard was applied in this assay. M: molecular ion.

References

Gavidia, I., Perez-Bermudez, P. and Seitz, H.U. (2002). Cloning and expression of two novel aldo-keto reductases from *Digitalis purpurea* leaves. *Eur. J. Biochem.* 269, 2842-2850

Yamauchi, Y., Hasegawa, A., Taninaka, A., Mizutani, M. and Sugimoto, Y. (2011). NADPH-dependent reductases involved in the detoxification of reactive carbonyls in plants. *J. Biol. Chem.* 286, 6999-7009
