# Supplementary material for: Diversified glucosinolate metabolism: biosynthesis of hydrogen cyanide and of the hydroxynitrile glucoside alliarinoside in relation to sinigrin metabolism in Alliaria petiolata
Source: Front Plant Sci. 2015 Oct 31;6:926. doi: 10.3389/fpls.2015.00926 (PMC4628127; doi:10.3389/fpls.2015.00926)
Supplement: Supplementary file 6 [file Image6.PDF]

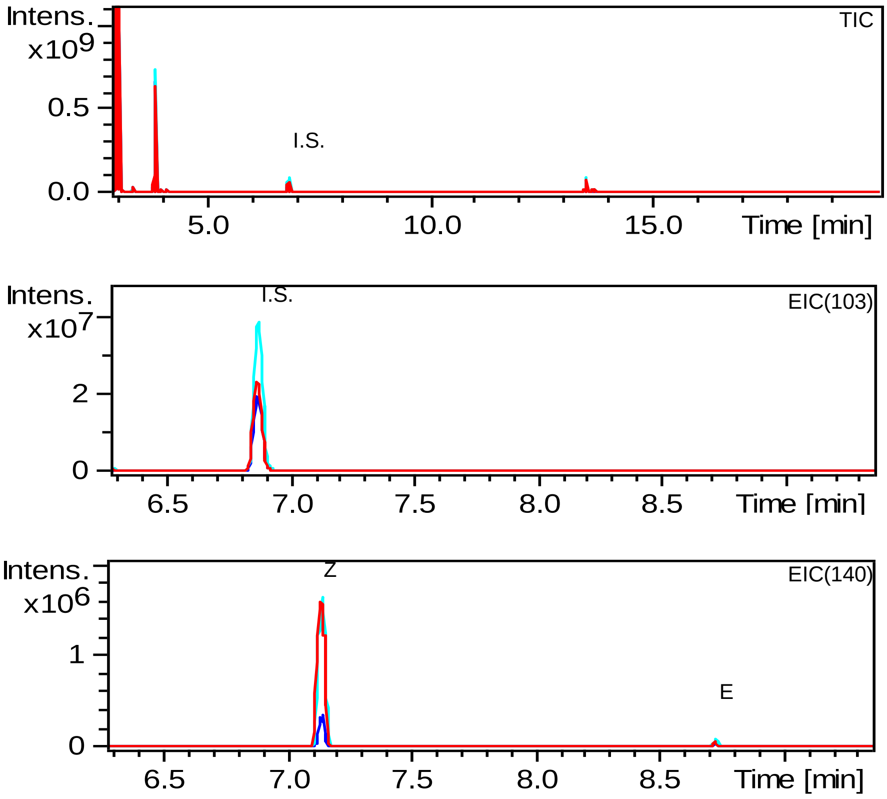

Figure S6: Production of (*Z*)- and (*E*)-4-hydroxy-2-butenenitrile (compounds 12 and 13) by soluble enzymes from endogenous substrate.

The content of (*Z*)-4-hydroxy-2-butenenitrile (Z) and (*E*)-4-hydroxy-2-butenenitrile (E) was determined by GC-MS in assays where soluble enzyme samples were incubated without cofactors (dark blue trace), a mixture of the cofactors NADPH, 2-oxoglutarate, ascorbate and catalase (red trace) or the cofactor mix supplemented with $FeSO_4$ (cyan trace). The peak intensity in EIC of *m*/*z* 140 (TMS-derivatives of E and Z) relative to EIC of m/z 103 (internal standard (I.S.), benzonitrile) showed that all soluble enzyme samples contained small amounts of Z and minute amounts of E. Furthermore, addition of cofactors resulted in 5- and 2-fold increase in the relative peak area of Z and E, respectively (red compared to dark blue trace). As no substrate was added to any of the samples shown, the small amounts of Z and E were produced cofactor-dependently from residual endogenous substrate in the desalted soluble enzyme fraction. Search for relevant masses did not reveal any candidate peaks and consequently the precursor of Z and E remains unidentified. Addition of $FeSO_4$ did not increase the content of Z or E (cyan trace) indicating that ferrous iron-dependent 2-ODD activity was not involved.
