# Supplementary material for: Diversified glucosinolate metabolism: biosynthesis of hydrogen cyanide and of the hydroxynitrile glucoside alliarinoside in relation to sinigrin metabolism in Alliaria petiolata
Source: Front Plant Sci. 2015 Oct 31;6:926. doi: 10.3389/fpls.2015.00926 (PMC4628127; doi:10.3389/fpls.2015.00926)
Supplement: Supplementary file 7 [file Image7.PDF]

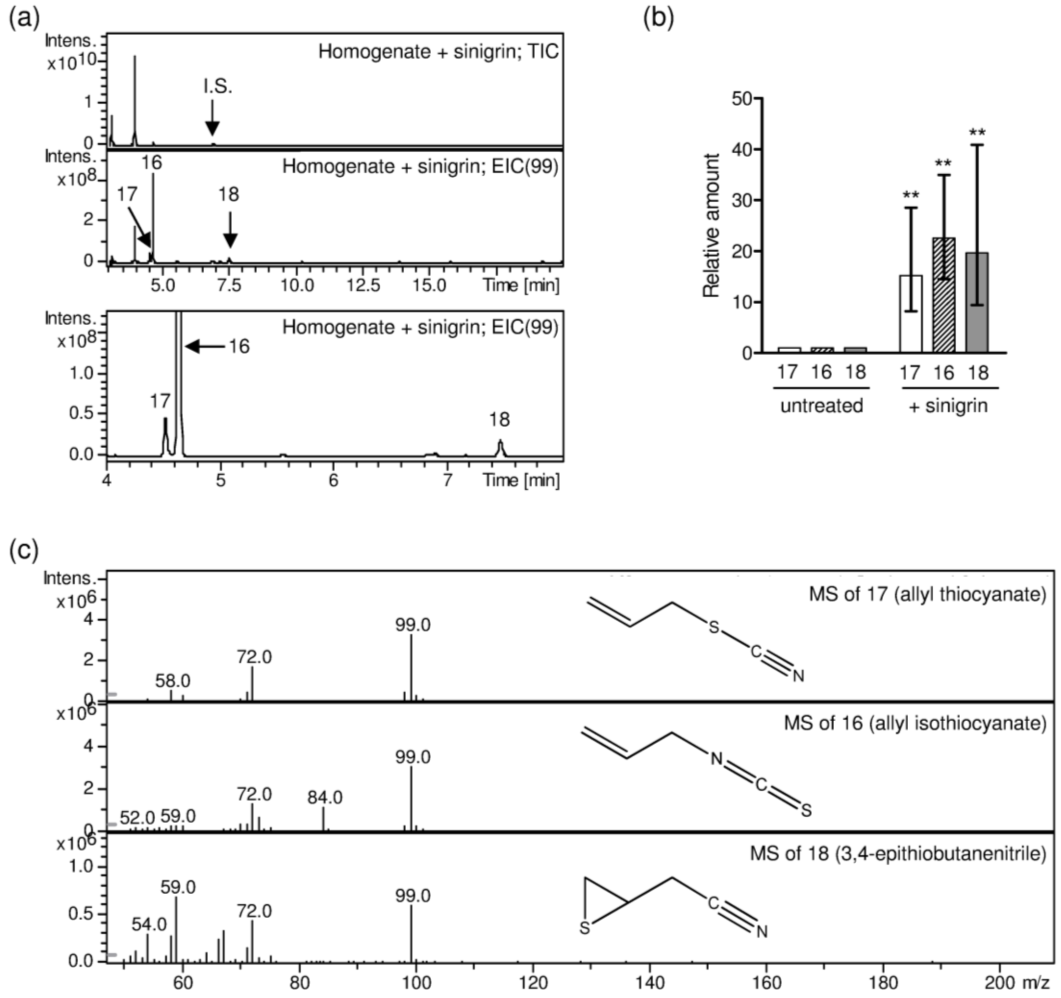

Figure S7: Allyl isothiocyanate (16), allyl thiocyanate (17) and 3,4-epithiobutanenitrile (18) are produced from sinigrin (15) in leaf homogenate. Leaf homogenate with or without exogenously added sinigrin was incubated and analysed by GC-MS. (a) Search for compounds of *m*/*z* 99 showed the presence of compounds **16**, **17** and **18**. The retention times and mass spectra (c) were compared to those in Wiley 9th Edition/ NIST 2011 MS database and published mass spectra in addition to an authentic standard of allyl isothiocyanate (Al-Gendy and Lockwood, 2003; Slater, 1992; Spencer and Daxenbichler, 1980). Based on this, the compounds were identified as allyl thiocyanate (**17**), allyl isothiocyanate (**16**) and 3,4-epithiobutanenitrile (**18**). (b) Addition of sinigrin to the homogenate significantly increased the amount of the three compounds compared to untreated samples. Data are shown as average peak areas of samples treated with sinigrin (right) relative to average

peak areas in the untreated samples (left), all set to 1. The significance was shown by a one-tailed, paired *t*-test of log_{10}-transformed data from three biological replicates. Processing of the data was performed as described for statistical analysis of the (*Z*)-4-hydroxy-2-butenenitrile content following sinigrin addition. The column graph shows back-transformed means ± SD. **: ($P<0.01$); I.S.: internal standard (benzonitrile, *m*/*z* 103); TIC: total ion chromatogram; EIC: extracted ion chromatogram.

These results show that addition of sinigrin to leaf homogenate yielded the common glucosinolate degradation product allyl isothiocyanate as well as allyl thiocyanate and 3,4-epithiobutanenitrile. Production of the latter two is likely to involve the thiocyanate-forming protein, ApTFP1, recently identified in *A. petiolata* (Kuchernig et al., 2012).

References

Al-Gendy, A.A. and Lockwood, G.B. (2003). GC-MS analysis of volatile hydrolysis products from glucosinolates in *Farsetia aegyptia* var. ovalis. *Flavour Frag. J.* 18, 148-152

Kuchernig, J., Burow, M. and Wittstock, U. (2012). Evolution of specifier proteins in glucosinolate-containing plants. *BMC Evol. Bio.* 12, 127

Slater, G. (1992). Allyl thiocyanate - Natural product or artifact in Crucifer extracts? *Chromatographia*, 34, 461-467

Spencer, G.F. and Daxenbichler, M.E. (1980). Gas chromatography-mass spectrometry of nitriles, isothiocyanates and oxazolidinethiones derived from cruciferous glucosinolates. *J. Sci. Food Agri.* **31**, 359-367
