# Supplementary material for: Diversified glucosinolate metabolism: biosynthesis of hydrogen cyanide and of the hydroxynitrile glucoside alliarinoside in relation to sinigrin metabolism in Alliaria petiolata
Source: Front Plant Sci. 2015 Oct 31;6:926. doi: 10.3389/fpls.2015.00926 (PMC4628127; doi:10.3389/fpls.2015.00926)
Supplement: Supplementary file 8 [file Image8.PDF]

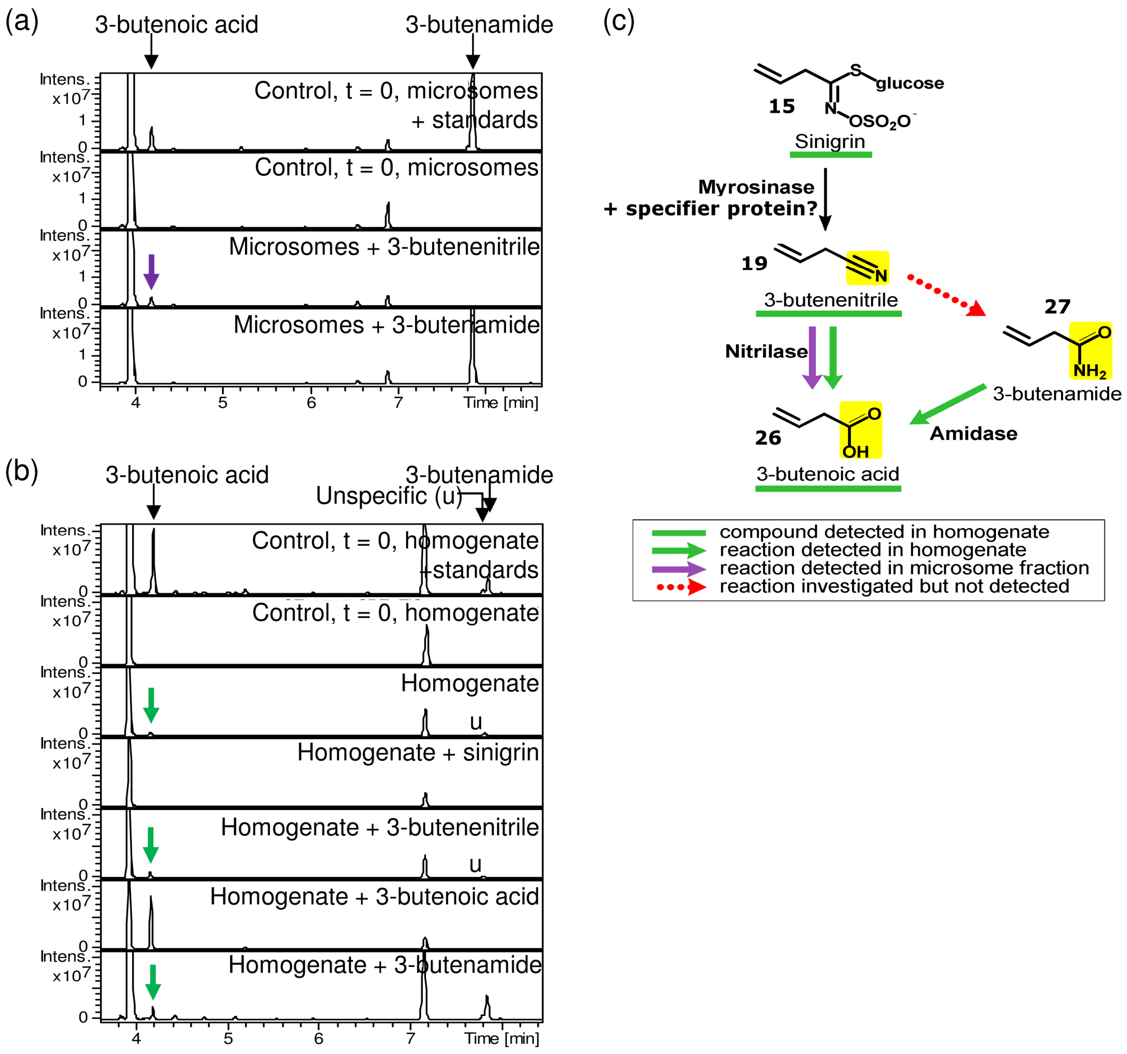

Figure S8: Nitrilase activity in homogenate and microsomal extracts of *A. petiolata* catalyses conversion of 3-butenenitrile (19) into 3-butenoic acid (26).

3-Butenenitrile was incubated with microsomes (panel a) or leaf homogenates (panel b) of *A. petiolata* and products formed were analysed by GC-MS using direct injection. EIC of TMS-derivatives of 3-butenoic acid (*m*/*z* 143) (**26**) and 3-butenamide (*m*/*z* 142) (**27**) are depicted. Panel c: Schematic overview of the monitored enzyme conversions. The relative amount of 3-butenoic acid increased, when 3-butenenitrile or 3-butenamide was added as substrate to the homogenate. u: A minor peak of a substrate-unspecific compound observed at a retention time very close to 3-butenamide (panel b).
