# Supplementary material for: Diversified glucosinolate metabolism: biosynthesis of hydrogen cyanide and of the hydroxynitrile glucoside alliarinoside in relation to sinigrin metabolism in Alliaria petiolata
Source: Front Plant Sci. 2015 Oct 31;6:926. doi: 10.3389/fpls.2015.00926 (PMC4628127; doi:10.3389/fpls.2015.00926)
Supplement: Supplementary file 9 [file Image9.PDF]

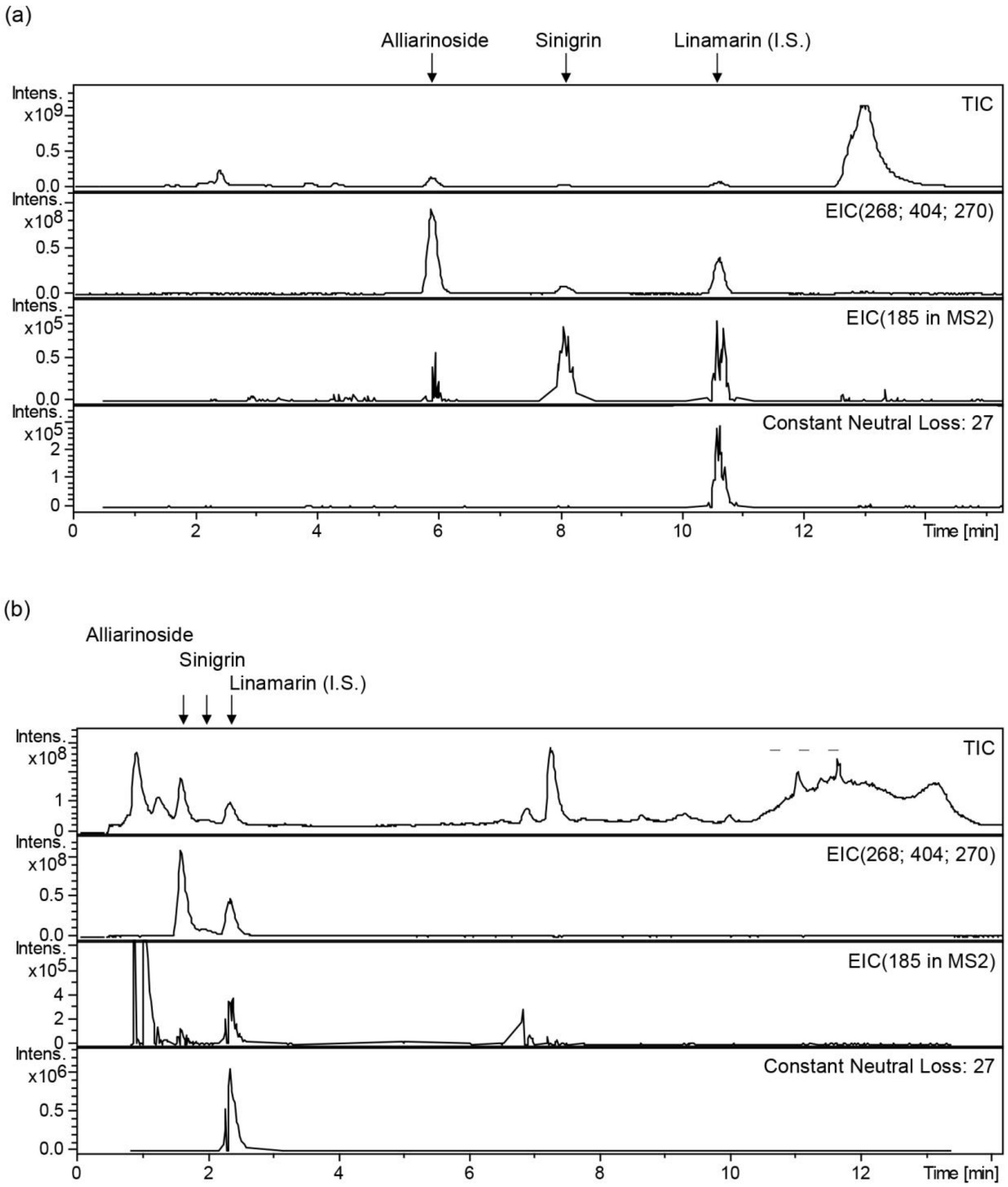

Figure S9: Searching for cyanogenic glucosides in *A. petiolata*.

Extracts of *A. petiolata* foliage were analysed by two LC-MS methods: One optimized for separation of the very polar alliarinoside (a) and a standard method for detection of glucosides (b) (Method S3). A constant neutral loss of 27 (HCN) diagnostic for cyanogenic glucosides in our LC-MS system was detected from the cyanogenic glucoside linamarin (*m*/*z* 270), which was applied as internal standard (I.S.), but from no endogenous compounds. The characteristic MS2 fragment ion of *m*/*z* 185 from glucosides ($[Glc + Na - H_2O]^+$) was detected from alliarinoside (**14**) (*m*/*z* 268), sinigrin (**15**) (*m*/*z* 404) and linamarin. The *m*/*z* 185 fragment detected at 6.8 min (panel b) originated from *m*/*z* 409. The accurate mass of *m*/*z* 409 suggests this to be the sodium adduct of sinapoyl glucose ($C_{17}H_{22}NaO_{10}$, error: 0.6 ppm), a common Brassicaceae product from the shikimate/

phenylpropanoid pathway involved in UV protection (Milkowski and Strack, 2010). No further attempts were made to identify this compound, which did not yield a constant neutral loss of 27. In conclusion, the metabolite profiles of *A. petiolata* leaves did not give any indications of presence of cyanogenic glucosides.

References

Milkowski, C. and Strack, D. (2010). Sinapate esters in brassicaceous plants: biochemistry, molecular biology, evolution and metabolic engineering. *Planta* 232, 19-35
