# Supplementary material for: Diversified glucosinolate metabolism: biosynthesis of hydrogen cyanide and of the hydroxynitrile glucoside alliarinoside in relation to sinigrin metabolism in Alliaria petiolata
Source: Front Plant Sci. 2015 Oct 31;6:926. doi: 10.3389/fpls.2015.00926 (PMC4628127; doi:10.3389/fpls.2015.00926)
Supplement: Supplementary file 10 [file Image10.PDF]

(a)

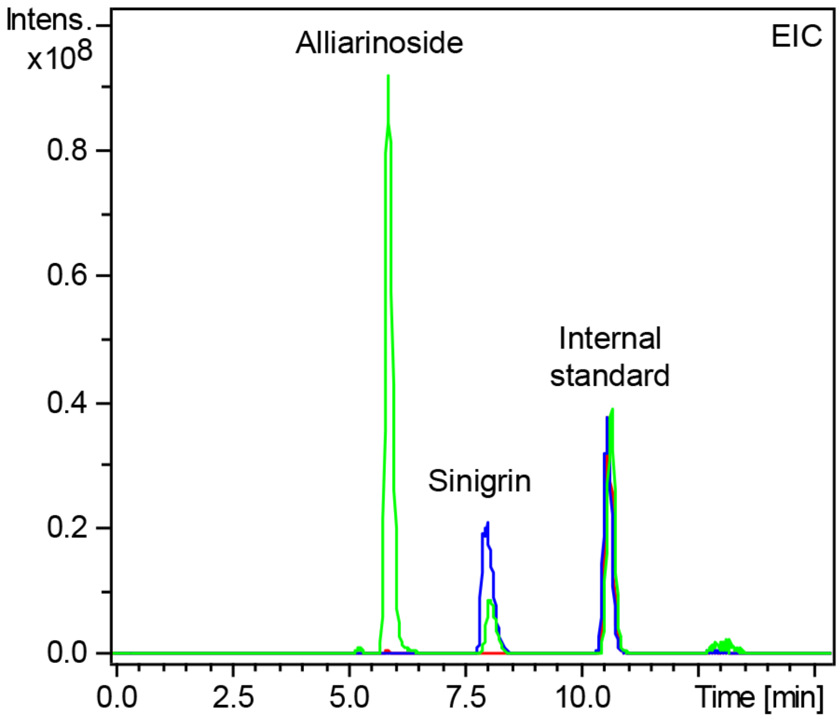

(b)

		Alliarinoside [nmol mg FW^{-1}]	Sinigrin [nmol mg FW^{-1}]
green	*A. petiolata*	15.1	5.0
blue	*B. juncea*	0	7.4
red	*A. thaliana*	0	0

Figure S10: Quantification of alliarinoside (14) and sinigrin (15) by LC-MS. Extracts of foliage of *A. petiolata* (green), *B. juncea* (blue) and *A. thaliana* Col-0 wt (red) were analysed by LC-MS for their concentration of alliarinoside and sinigrin. (a) The extracted ion chromatograms (EIC) of the sodium adduct of alliarinoside (*m*/*z* 268), sinigrin (*m*/*z* 404) and internal standard linamarin (*m*/*z* 270) confirmed that sinigrin was present in *A. petiolata* and *B. juncea*. Alliarinoside was present in *A. petiolata* only. (b) Concentrations were determined in extracts of leaves harvested from three individual plants.
