# Supplementary material for: Diversified glucosinolate metabolism: biosynthesis of hydrogen cyanide and of the hydroxynitrile glucoside alliarinoside in relation to sinigrin metabolism in Alliaria petiolata
Source: Front Plant Sci. 2015 Oct 31;6:926. doi: 10.3389/fpls.2015.00926 (PMC4628127; doi:10.3389/fpls.2015.00926)
Supplement: Supplementary file 11 [file Image11.PDF]

(a)

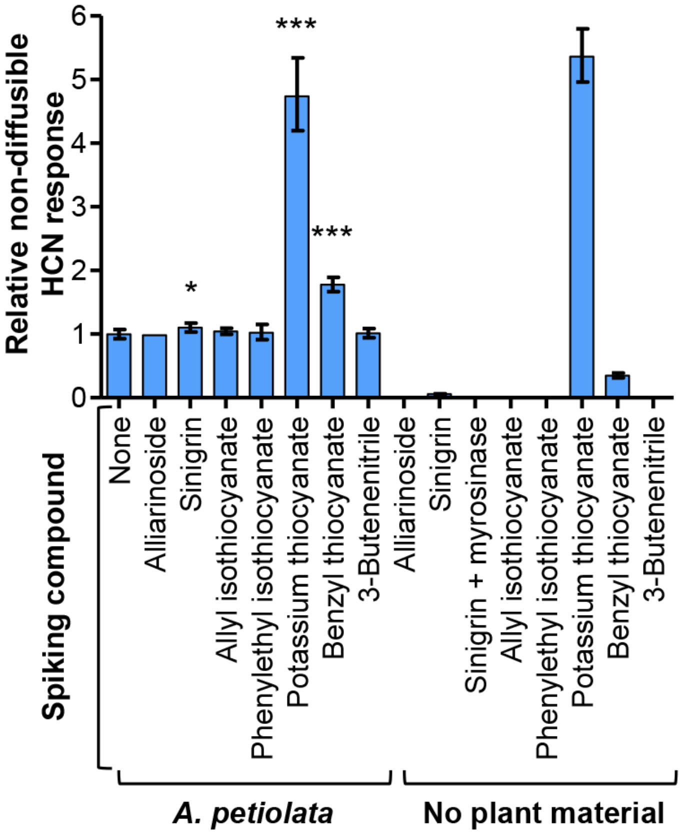

6
5
4
3
2
1
0
Relative non-diffusible HCN response
***
***
*
Spiking compound
None
Alliarinoside
Sinigrin
Allyl isothiocyanate
Phenylethyl isothiocyanate
Potassium thiocyanate
Benzyl thiocyanate
3-Butenenitrile
Alliarinoside
Sinigrin
Sinigrin + myrosinase
Allyl isothiocyanate
Phenylethyl isothiocyanate
Potassium thiocyanate
Benzyl thiocyanate
3-Butenenitrile
A. petiolata
No plant material

(b)

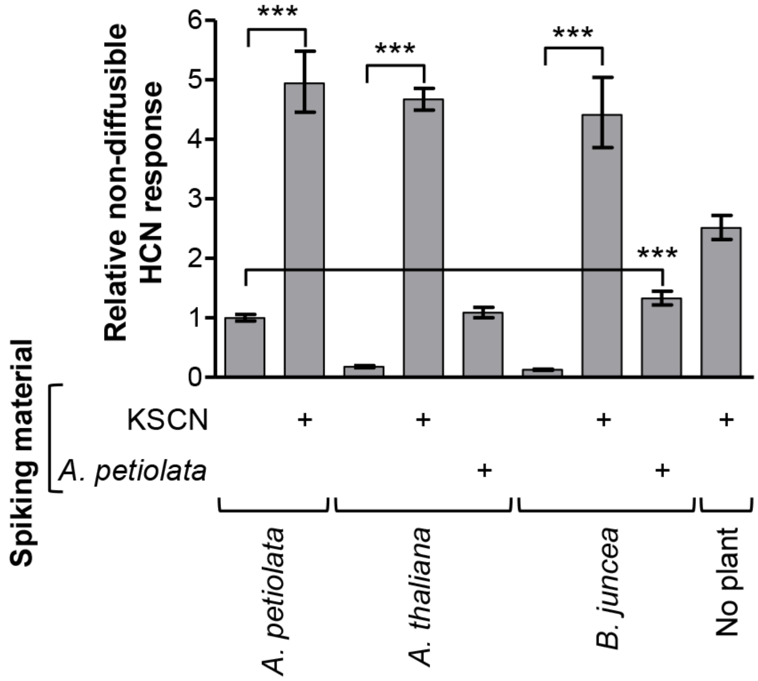

6
5
4
3
2
1
0
Relative non-diffusible HCN response
***
***
***
***
Spiking material
KSCN
A. petiolata
+
A. petiolata
A. thaliana
B. juncea
No plant

Figure S11: Inorganic and organic thiocyanate interfere in the König reaction for HCN detection when the diffusion step is not included.

The colorimetric response in the König reaction-based method for HCN detection was quantified in leaf homogenate suspensions hereby assessing the presence of non-diffusible reactants providing a colour reaction, here termed the non-diffusible HCN response. The corresponding analyses of the diffusible HCN response obtained in the alkaline trap are shown in figure 6 and figure 7.

a) The effect of spiking *A. petiolata* leaf homogenate with alliarinoside (**14**) (final concentration: 8 nmol mg FW^{-1}), sinigrin (**15**) or available glucosinolate degradation products (final concentration: 10 nmol mg FW^{-1}) was quantified relative to the mean of untreated samples. The non-diffusible HCN response increased significantly (one-tailed unpaired *t*-test of log_{10}-transformed ratios), when *A. petiolata* leaf homogenate was added exogenous sinigrin (*: $P<0.05$), KSCN or benzyl thiocyanate (***: $P<0.0001$). When no plant material was present, KSCN and to a lesser degree benzyl thiocyanate gave a non-diffusible colour response. This demonstrates that inorganic and organic thiocyanates interfere in the König reaction-based method for HCN detection and highlights the importance of including a diffusion step prior to HCN quantification by trapping volatile HCN in an alkaline trap as described in the experimental procedures (leaf suspension: pH 6, alkaline trap: pH 14, HCN $pK_a=9.2$, HSCN $pK_a=0.9$). The small increase in the non-diffusible HCN response in sinigrin-spiked samples compared to untreated samples was most likely due to allyl thiocyanate formation following sinigrin degradation as observed in *A. petiolata* homogenate (figure S7).

The non-diffusible response of untreated samples was equivalent to a HCN potential of 2.1 ± 0.2 nmol mg FW^{-1} (mean ± SD). This was 5 fold higher than the diffusible HCN response of untreated samples (0.4 ± 0.02 nmol mg FW^{-1}).

b) The non-diffusible HCN response in the König-reaction was determined in suspensions of leaves from *A. thaliana* and *B. juncea* relative to the non-diffusible HCN response from *A. petiolata*. Addition of KSCN demonstrated that in all three tested *Brassicaceae* species exogenous KSCN interfered in the König reaction (one-tailed unpaired *t*-test of log_{10}-transformed ratios, ***: P<0.0001)

Bars represent back-transformed mean ± SD (*n*=4, except alliarinoside: *n*=2).
