# Supplementary material for: Diversified glucosinolate metabolism: biosynthesis of hydrogen cyanide and of the hydroxynitrile glucoside alliarinoside in relation to sinigrin metabolism in Alliaria petiolata
Source: Front Plant Sci. 2015 Oct 31;6:926. doi: 10.3389/fpls.2015.00926 (PMC4628127; doi:10.3389/fpls.2015.00926)
Supplement: Supplementary file 12 [file DataSheet1.DOCX]

## Methods S1: Chemical Synthesis

All reactions were monitored by TLC on aluminium sheets coated with silica gel 60F254 (0.2 mm thickness, Merck) and the components present were detected by charring with 10% H_2_SO_4_ in MeOH. Column chromatography was carried out using silica gel 60 (particle size 0.040-0.063 mm, 230-400 mesh ASTM, Merck). Solvent extracts were dried with anhydrous MgSO_4_ unless otherwise specified. The ^1^H and ^13^C NMR spectra were recorded on a Bruker Avance 400 spectrometer at 400 and 101 MHz, respectively. CDCl_3_ was used as solvent (unless otherwise indicated), δH values are relative to internal TMS and δC values are referenced to the solvent [δC (CDCl_3_) = 77.0; δC (CD_3_OD) = 49.5; δC (Acetone-*d*6) = 206 ppm].

#### 2-Hydroxy-4-(methylthio)butanenitrile (5) (Figure 9)

A mixture of 3-(methylthio)propionaldehyde (**21**) (1.0 ml, 10 mmol), solid LiClO_4_ (1.06 g, 10 mmol) and TMSCN (1.9 ml, 15.2 mmol) was stirred at room temperature (r.t.) for 2 h. CH_2_Cl_2_ was added to the reaction mixture and LiClO_4_ was filtered off. The organic layer was washed with water and dried over anhydrous Na_2_SO_4_. Solvent was removed by a rotary evaporator to obtain almost pure crude product of *O*-(trimethylsilyl)-2-hydroxy-4-(methylthio)butanenitrile. Tetrahydrofuran (THF, 30 ml) and HCl (20 ml, 3M) were added to the crude product obtained. The mixture was heated at 65 °C (oil bath temperature) for 1 h. The solution was poured into a separatory funnel and 30 ml of water was added. The aqueous phase was separated and back-extracted with three 100 ml portions of diethyl ether. The ethereal extracts are combined with the tetrahydrofuran solution and dried, filtered, and solvent was removed by evaporation on a rotary evaporator to give pure **5** as dark red coloured liquid (1.1 g, 8.4 mmol, 84%). ^1^H-NMR (400 MHz, CDCl_3_): δ = 2.17-2.10 (m, 5 H, CH_3_S and H-3), 2.72 (m, 2 H, H-4), 3.78 (br.s, 1 H, OH), 4.72 (m, 1 H, H-1); ^13^C-NMR (100 MHz, CDCl_3_): δ = 15.4 (CH_3_S), 29.1 (C-4), 33.7 (C-3), 60.0 (C-2), 119.7 (CN). These data were consistent with those previously reported (Ziegler *et al.*, 1990; Ognyanov *et al.*, 1991) for **5**.

#### 3-(tert-Butyldiphenylsilyloxy)-1-propene (23) (Figure 10)

*tert*-Butyldiphenylchlorosilane (20.6 ml, 80.5 mmol, 1.1eq) was added to a stirred solution of allyl alcohol (**22**) (5.0 ml, 73.2 mmol), trimethylamine (TEA; 12.3 ml, 87.8 mmol, 1.2 eq), and N,N-dimethylaminopyridine (DMAP; 1.0 g, 8.2 mmol) in CH_2_Cl_2_ (20 ml) at r.t. under argon. Following stirring at r.t. for 1 h, the reaction mixture was diluted with excess ethyl acetate (200 ml) and the organic phase was washed with ice-cold 1 N HCl (3 x 100 ml), water (3 x 50 ml), sodium hydrogen carbonate aq. sat solution (3 x 50 ml), brine (50 ml), dried and evaporated to dryness. The obtained residue was chromatographed on silica (150 g) with 0-5 % diethyl ether in *n*-pentane to afford pure **23** as colourless liquid (21.0 g, 97 %). ^1^H-NMR (400 MHz, CD_3_COCD_3_): δ = 1.06 (s, 9 H, C*(CH_3_)_3_*), 4.25 (m, 2 H, CH_2_), 5.12 (ddd, 1 H, J = 10.5, 4.1, 1.9 Hz, H-3), 4.40 (dd, 1 H, *J* = 17.3, 4.1, 2.0, Hz, H-3), 5.97 (ddt, 1 H, *J* = 17.0, 8.5, 4.2 Hz, H-2), 7.44 (m, 6 H, H-arom.), 7.72 (m, 4 H, H-arom.); ^13^C-NMR (100 MHz, CD_3_COCD_3_): δ = 19.8 (*C*(CH_3_)_3_), 27.2 (3 x CH_3_), 65.3 ( C-1), 114.1 (C-3), 134.3 (C-2), 138.0, 136.2, 130.6 and 128.6 (12 x C, C-arom.). These data were consistent with those previously reported (Saygili *et al.*, 2001) for **23**.

#### (R,S)-(O-tert-Butyldiphenylsilyloxymethyl)oxirane (24) (Figure 10)

A solution of **23** (16.8 g, 56.6 mmol) and m-chloroperbenzoic acid 70 % (m-CPBA; 28.0 g, 113.2 mmol) in dry 1,2-dichloroethane (DCE; 100 ml) was refluxed for 30 min. The reaction mixture was kept overnight at 4-6°C, then filtered , concentrated and chromatographed on silica (300 g) with 0-10 % diethyl ether in *n*-pentane to afford pure **24** as a colourless liquid (16.8 g, 95%). ^1^H-NMR (400 MHz, CD_3_COCD_3_): δ = 1.05 (s, 9 H, C*(CH_3_)_3_*), 2.59 (dd, 1 H, *J* = 5.3, 2.6 Hz, H-3), 2.71 (dd, 1 H, *J* = 5.3, 4.1 Hz, H-3), 3.154-3.117 (m, 1 H, H-2), 3.67 (dd, 1 H, J = 12.0, 5.3 Hz, H-1), 3.94 (dd, 1 H, J = 11.7, 2.9 Hz, H-1), 7.50-7.40 (m, 6 H, H-arom.), 7.76-7.70 (m, 4 H, H-arom.): ^13^C-NMR (100 MHz, CD_3_COCD_3_): δ = 19.8 (*C*(CH_3_)_3_), 27.1 (3 x CH_3_), 44.2 ( C-3), 52.6 (C-2), 65.6 (C-1), 136.3, 134.1, 130.7 and 128.7 (12 x C, C-arom.). These data were consistent with those previously reported (Gao *et al.*, 1987; Dixon *et al.*, 2002; Betancort *et al.*, 2003; Agrawal *et al.*, 2006; Huckins *et al.*, 2007; Dias and Ferreira, 2012; Ekhato and Palazzolo, 1998) for **24**.

#### 3,4-Dihydroxybutanenitrile (11) (Figure 10)

A mixture of epoxide **24** (1.6 g, 5.0 mmol), solid LiClO_4_.3 H_2_O (0.08 g, 5.0 mmol, 10.0 mol %) and TMSCN (0.75 ml, 5.0 mmol) was heated at 85 °C for 1 h under nitrogen. The reaction mixture was cooled to r.t., CH_2_Cl_2_ was added and LiClO_4_ was filtered off. The organic layer was washed with water, dried over anhydrous Na_2_SO_4_ and evaporated. Tetra-*n*-butylammonium fluoride (TBAF; 1M in THF: 20.0 ml, 20.0 mmol) was added to a stirred solution of the residue obtained in THF (10.0 ml) at r.t. and stirring continued for 30 min. The reaction mixture was diluted with CH_2_Cl_2_ and filtered over silica gel, washed with CH_2_Cl_2_ until no UV active products were detected. The silica was washed several times with 50% MeOH in CH_2_Cl_2_, the collected filtrate was evaporated and the residue obtained was chromatographed on silica (60 g) with 0-10% MeOH in CH_2_Cl_2_ as gradient eluent to obtain pure **11** as yellow liquid (0.44 g, 88%). ^1^H-NMR (400 MHz, CD_3_OD): δ = 2.57 (dd, 1 H, *J* = 16.9, 7.0 Hz, H_b_-2), 2.69 (dd, 1 H, *J* = 16.9, 4.7 H, H_a_-2), 3.32-3.30 (m, OH-3 and OH-4), 3.49 (dd, 1 H, *J* =11.1, 5.8 Hz, H_b_-4), 3.57 (dd, 1 H, *J* = 11.1, 5.3 Hz, H_a_-4), 3.88 (quintet, 1 H, *J* _A,B_ = 5.6 Hz, H-3); ^13^C-NMR (100 MHz, CDCl_3_): δ = 22.9 (C-2), 65.7 (C-3), 69.1 (C-4), 119.2 (CN).

#### (E/Z)-4-tert-Butyldiphenylsilyloxy-2-butenenitrile (25) (Figure 10)

A mixture of epoxide **24** (3.1 g, 10.0 mmol), solid LiClO_4_.3H_2_O (0.16 g, 5.0 mmol, 10.0 mol %) and TMSCN (1.5 ml, 5.0 mmol) was heated at 85 °C for 1 h under nitrogen. The reaction mixture was cooled to r.t. and pyridine (20 ml) and POCl_3_ (3.0 ml, 32.2 mmol) were added. The reaction mixture was heated at 85 °C for 1 h under nitrogen then cooled to r.t. and diethyl ether (200 ml) was added. The mixture was filtered through a layer of sand and silica gel. The silica gel was washed several times with diethyl ether (5 x 25 ml), and the collected filtrate was washed thoroughly with saturated aq NaHCO_3_ (3 x 50 ml), water (3 x 50 ml), and brine (50 ml), dried and evaporated to dryness. The residue was chromatographed on silica gel (150 g) with 0-3 % ethyl acetate/ *n*-pentane as eluent to give **25** (2.8g, 87%) as colourless liquid.

*(E)*-Isomer: ^1^H-NMR (400 MHz, CD_3_COCD_3_): δ = 1.08 (s, C*(CH_3_)_3_*), 4.44 (t, *J* = 2.8 Hz, -OCH_2_), 5.99 (dt, 0.6 H, *J* = 16.1, 2.5 Hz, H-2), 6.96 (dt, 0.6 H, *J* = 16.4, 3.1 Hz, H-3), 7.52-7.43 (m, H-arom.), 7.75-7.67 (m, H-arom.): ^13^C-NMR (100 MHz, CD_3_COCD_3_): δ = 19.7 (*C*(CH_3_)_3_), 27.0 (3 x CH_3_), 63.8 ( C-4), 98.6 (C-2), 118.1 (CN), 136.1, 133.4,130.8 and 128.7 (12 x C, C-arom.), 154.2 (C-3).

*(Z)*-Isomer: ^1^H-NMR (400 MHz, CD_3_COCD_3_): δ = 1.07 (s, C*(CH_3_)_3_*), 4.53 (dd, *J* = 5.6, 1.8 Hz, -OCH_2_), 5.65 (dt, 0.32 H, *J* = 11.4, 1.8 Hz, H-2), 6.75 (dt, 0.32 H, *J* = 11.2, 5.6 Hz, H-3), 7.52-7.43 (m, H-arom.), 7.75-7.67 (m, H-arom.): ^13^C-NMR (100 MHz, CD_3_COCD_3_): δ = 19.6 (*C*(CH_3_)_3_), 27.0 (3 x CH_3_), 63.6 ( C-4), 99.7 (C-2), 116.0 (CN), 136.2, 133.5,130.8 and 128.7 (12 x C, C-arom.), 153.0 (C-3).

#### (E)-4-Hydroxy-2-butenenitrile (13) (Figure 10)

TBAF (1M in THF: 10.0 ml, 10.0 mmol) was added to a stirred solution of **25** (1.6 g, 5.0 mmol) in THF (10.0 ml) at r.t. and stirring continued for 30 min. The reaction mixture was diluted with CH_2_Cl_2_ and filtered over silica gel, washed with CH_2_Cl_2_ until no UV active products were detected. The silica was washed several times with diethyl ether and the collected filtrate was carefully concentrated and chromatographed on silica (40 g) with 10-50% diethyl ether in *n*-pentane as gradient eluent to obtain pure **13** as colourless liquid (0.66 g, 79%). ^1^H-NMR (400 MHz, CDCl_3_): δ = 2.44 (m, 2-H, OCH_2_), 5.74 (dt, 1 H, *J* = 16.4, 2.3 H, H-2), 6.85 (dt, 1 H, *J* =16.1, 3.5 Hz, H-3); ^13^C-NMR (100 MHz, CDCl_3_): δ = 61.6 (C-4), 98.6 (C-2), 117.4 (CN), 153.6 (C-3). These data were consistent with those previously reported (Fleming *et al.*, 2001; Nudelman and Keinan, 1982) for **13**.
